# Fracture Dynamics in Silicon Anode Solid-State Batteries

**DOI:** 10.1021/acsenergylett.4c02800

**Published:** 2024-11-26

**Authors:** Douglas Lars Nelson, Stephanie E. Sandoval, Jaechan Pyo, Donald Bistri, Talia A. Thomas, Kelsey Anne Cavallaro, John A. Lewis, Abhinav S. Iyer, Pavel Shevchenko, Claudio V. Di Leo, Matthew T. McDowell

**Affiliations:** † School of Materials Science and Engineering, Georgia Institute of Technology, Atlanta, Georgia 30332, United States; ‡ Daniel Guggenheim School of Aerospace Engineering, Georgia Institute of Technology, Atlanta, Georgia 30332, United States; § George W. Woodruff School of Mechanical Engineering, Georgia Institute of Technology, Atlanta, Georgia 30332, United States; ∥ Advanced Photon Source, Argonne National Laboratory, Lemont, Illinois 60439, United States

## Abstract

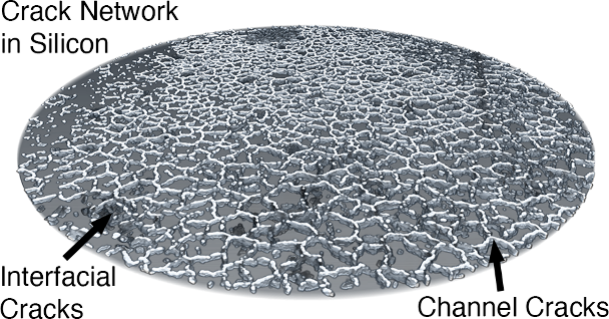

Solid-state batteries (SSBs) with silicon anodes could enable improved safety and energy density compared to lithium-ion batteries. However, degradation arising from the massive volumetric changes of silicon anodes during cycling is not well understood in solid-state systems. Here, we use *operando* X-ray computed microtomography to reveal micro- to macro-scale chemo-mechanical degradation processes of silicon anodes in SSBs. Mud-type channel cracks driven by biaxial tensile stress form across the electrode during delithiation. We also find detrimental cracks at the silicon/solid electrolyte interface that form due to local reaction competition between neighboring domains of different sizes. Continuum phase-field damage modeling quantifies stress-driven channel cracking and shows that the lithiated silicon stress state is critical for determining the extent of interfacial fracture. This work reveals the mechanisms that govern SSBs compared to conventional lithium-ion batteries and provides guidelines for engineering chemo-mechanically resilient electrodes for high-energy batteries.

Silicon shows great promise as a lithium battery anode material due to its high specific capacity (3579 mAh g^–1^),^1^ natural abundance,^2^ low cost,^2^ environmental friendliness,^3^ and ease of manufacturing.^4,5^ However, its use in conventional lithium-ion batteries has been limited because of massive volumetric changes during cycling (>300%).^1,6^ Expansion and contraction during lithiation/delithiation cause continuous growth of the solid-electrolyte interphase (SEI) as the liquid electrolyte reacts with newly exposed surface each cycle.^6−9^ In SSBs, this continuous SEI growth and associated capacity fade are largely mitigated since the electrolyte does not flow into cracks.^3,5,10−12^ Due to limited SEI growth, micron-scale silicon particles can be used within SSB anodes,^3,10^ whereas nanosized silicon with complex engineered structure is needed to minimize SEI growth in liquid-electrolyte systems.^7,9,13^ Recent studies have achieved stable cycling of silicon-based SSBs at relatively high stack pressure, highlighting the potential of silicon as a SSB anode.^10,14−17^

Despite recent promising performance, the fundamental understanding of and control over the dynamic evolution of silicon anodes in SSBs has yet to be realized.^3,5^ The nanoscale reaction mechanisms of silicon with lithium have previously been investigated with a variety of *in situ* experiments, including X-ray diffraction,^18,19^ transmission electron microscopy,^20−22^ and nuclear magnetic resonance,^23,24^ and the behavior of silicon electrodes in lithium-ion batteries is fairly well understood.^25−27^ However, SSBs present a completely different electro-chemo-mechanical environment with different expected behavior. Morphology evolution and chemo-mechanical damage of silicon electrodes in SSBs have been observed *ex situ* with scanning electron microscopy (SEM) and focused-ion beam (FIB) SEM.^4,10,15−17,28^ Thick silicon electrodes operated under stack pressure in SSBs have been shown to exhibit a vertically oriented network of “mud-cracks” after delithiation,^4,10,17,28,29^ similar to silicon electrodes in liquid-electrolyte systems.^6,8,30,31^ While these observations provide a glimpse into the behavior of silicon electrodes, they fail to capture dynamic evolution and they can only provide a limited view of the silicon/solid-state electrolyte (SSE) interface (<100 μm). Additionally, *ex situ* characterization is destructive and is performed after removal of the stack pressure present during testing, which likely alters the interface from its true state. Thus, *operando* characterization is needed to relate structural evolution to electrochemical performance.

X-ray computed tomography (XCT) is a powerful technique for revealing the morphological evolution of materials and buried interfaces due to its broad field of view and nondestructive nature. XCT imaging has proven invaluable for observing the evolution of battery materials,^32−35^ and most efforts have focused on understanding the behavior of lithium metal anodes.^36−40^ XCT has also been used to characterize silicon electrodes for lithium-ion batteries.^41−43^ Recent investigations of composite silicon anodes in SSBs have investigated electrode additives and examined composite electrode evolution,^44,45^ but comprehensive investigation of degradation at the SSE/silicon interface is needed.

Here, we use *operando* XCT to investigate the structural evolution of 99.0% microparticle silicon anodes in SSBs with Li_6_PS_5_Cl (LPSC) solid-state electrolyte. Vertical mud-type channel cracks grow through the thickness of the silicon during delithiation, as driven by shrinkage-induced biaxial tensile stress. These cracks are observed to close near the SSE interface during partial relithiation. XCT analysis reveals newly observed interfacial fracture processes at the silicon/LPSC interface, in which interfacial cracks can form during both delithiation and relithiation. The formation of interfacial cracks is caused by the local morphological dynamics of neighboring silicon domains, indicating that electrode inhomogeneities can lead to interfacial contact loss and reduction of active electrode area. To quantify the links between stress and damage, a diffusion-deformation-damage continuum phase-field framework is developed^46,47^ which captures the dynamic formation of the observed mud-type channel cracks and demonstrates that crack spacing depends on electrode thickness/material properties. The formation of interfacial cracks is modeled in the presence of film imperfections, revealing the importance of initial stress state within the silicon in determining interfacial fracture. Together, these results provide new insight and strategies for engineering the chemo-mechanical properties of silicon electrodes, and they shed light on key mechanistic differences between emerging solid-state and conventional liquid-electrolyte battery systems.

We carried out *operando* synchrotron XCT with half cells in a custom cell housing with 2 mm internal diameter to enable transmission of X-rays (Figure 1a).^36,37,39^ The working electrode consisted of 99.0 wt % silicon microparticles slurry-cast on copper foil with an areal loading of 2.0 mg Si cm^–2^ (7.15 mAh cm^–2^), and the counter electrode was lithium metal. We chose this system because silicon electrodes with high active material loading have been shown to cycle well in SSBs and also can enable high energy densities.^10^ Composite silicon/SSE electrodes containing SSE also display interfacial fracture.^45^ The cylindrical cell was rotated during galvanostatic testing under X-ray illumination to collect projection images, which were then reconstructed into three-dimensional data sets of the cell stack with a voxel size of 1.4 μm (Figure 1a). An experiment with voltage curves shown in Figure 1b involved first collecting a 3D data set of the pristine cell, then lithiating the silicon electrode and collecting another 3D data set. *Operando* scans were then performed during further delithiation and relithiation of this cell, with 30 *operando* data sets collected during delithiation and 12 during lithiation at 15 min intervals. The slight divots in the *operando* voltage curves in Figure 1b occurred during X-ray exposure, as has been reported in prior work.^36^ While this small-scale XCT cell showed similar electrochemical behavior to 1 cm diameter anvil cells during the first cycle (Figure S1),^10,15,16^ the cutoff voltage during the *operando* relithiation portion was reached prematurely compared to anvil cells, resulting in lower capacity. This also resulted in accelerated capacity decay in the tomography cells with further cycling (Figure S1). The reasons for this are discussed subsequently.

**Figure 1:**
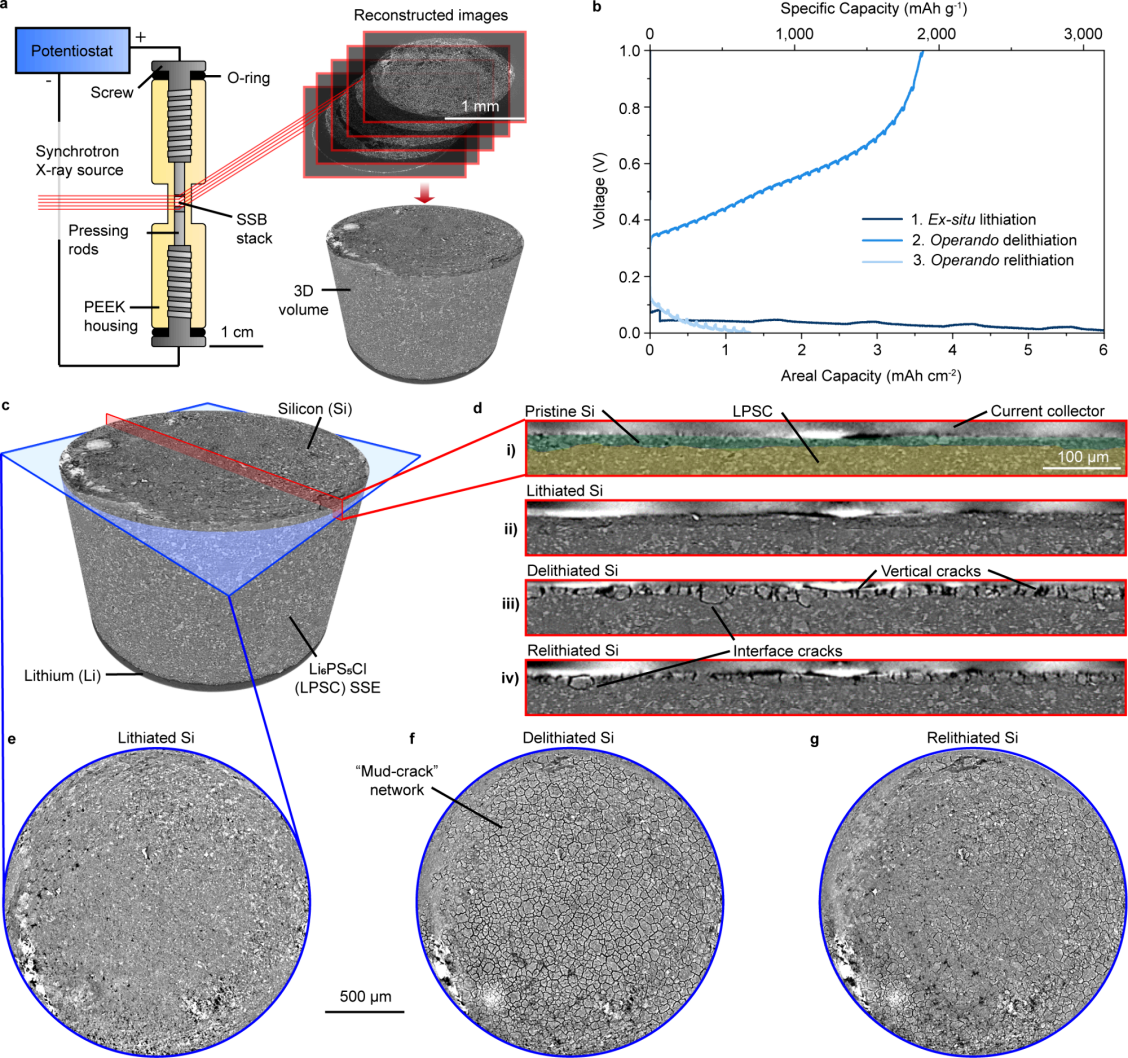
Experimental design and selected images from *operando* XCT. (a) Schematic representation of the *operando* XCT cell design, imaging, and image reconstruction procedure. (b) Galvanostatic voltage profile of a silicon half cell cycled during the *operando* XCT experiment at 0.5 mA cm^–2^ current density, 10 MPa stack pressure, and 25 °C. XCT images were collected before and after the first lithiation, and then every 15 min during delithiation and relithiation. (c) Reconstructed 3D rendering of the cell stack from XCT data, with different 2D slices highlighted. (d) Vertical cross-section images showing the silicon/LPSC interface in the (i) pristine, (ii) lithiated, (iii) delithiated, and (iv) relithiated states, with false-color overlay highlighting silicon and LPSC in (i). (e–g) Planar images from the midpoint of the silicon electrode in the (e) lithiated, (f) delithiated, and (g) relithiated states.

Figure 1c shows the reconstructed 3D volume obtained from the *operando* XCT experiment with two different 2D sections highlighted. One shows the silicon/LPSC interface, with 2D cross-sectional image slices at different times shown in Figure 1d. The other is oriented within the silicon electrode plane, and its 2D image slices are shown in Figure 1e-g. As seen in the cross-sectional images, the ∼10 μm thick pristine silicon electrode (Figure 1d(i)) increases in thickness after lithiation and exhibits slightly darker absorption contrast due to uptake of lithium (Figure 1d(ii)). Individual particulates in the pristine film are less distinct after lithiation, and the electrode is more continuous due to the volume expansion and merging into larger Li_*x*_Si particles. The merging of silicon particles to form continuous films during lithiation has been observed in previous studies^10,16,28^ and is qualitatively evident from the change in visual texture from rough and particulate in the pristine state (Figure 1d(i)) to smooth and conjoined in the lithiated state (Figure 1d(ii)). However, the resolution of these experiments (1.4 μm voxel size) does not allow for detailed analysis of particle merging or visibility of the binder material (PVDF). This limitation precludes analysis of very thin cracks (<1 μm) between silicon particles or at the Si/SSE interface, but the larger mud-type crack network and interfacial cracks occurring by the mechanisms detailed below are still resolvable. Some thin cracks are also visible in select locations of the lithiated electrode, but these are very few.

After delithiation, the entire electrode shows mud-type channel cracking, with vertical cracks forming between distinct domains of silicon (Figure 1d(iii) and Figure 1f). The average diameter of these domains is 29.5 μm ± 5.4 μm. Channel cracking has been observed in previous studies using thin film or dense silicon electrodes in liquid electrolytes, and it arises due to biaxial tension in the delithiated region under constraint from the remainder of the film.^6,30,31^ The average silicon domain size after one cycle is much larger than the initial average silicon particle size of 3.31 μm ± 1.92 μm (Figure S2), which further indicates that the Li_*x*_Si particles merge to become a continuous Li_*x*_Si film during lithiation. A few delamination-type interfacial cracks separating silicon domains from the LPSC are also visible in the cross section after delithiation (Figure 1d(iii)). After relithiation and volume expansion (Figure 1d(iv)), most interfacial cracks are closed, but one is visible in the cross-sectional image, isolating a silicon domain and leaving it unreacted. The planar view of the relithiated state (Figure 1g) shows a significant reduction in the number of channel cracks due to swelling of the silicon electrode. Since relithiation was only partial, not all cracks were completely closed, with some remaining open near the current collector interface (Figure 1d(iv)). Supplementary Video 1 shows the evolution of these reaction processes for the same view orientation seen in Figure 1e-g.

To observe the evolution of the crack network, we used image segmentation procedures to classify and sort fractured voxels from other phases based on intensity after applying an edge-preserving Gaussian blur filter (see Methods and SI Section S2). Figure 2a-c show renderings of the 3D crack network in the lithiated, delithiated, and partially relithiated states. Figure 2d shows a tilted view of the 3D crack network in the delithiated state, highlighting the channel cracks extending through the thickness of the silicon electrode. Figure 2e shows the same tilted view after partial relithiation. Interfacial cracks at the silicon/LPSC interface are also visible in both the delithiated and partially relithiated states; they appear as dark regions in Figure 2b-c that span single silicon domains within channel crack boundaries. There are more interfacial cracks after delithiation than relithiation, and very few cracks of either type are present in the lithiated state (Figure 2a).

**Figure 2:**
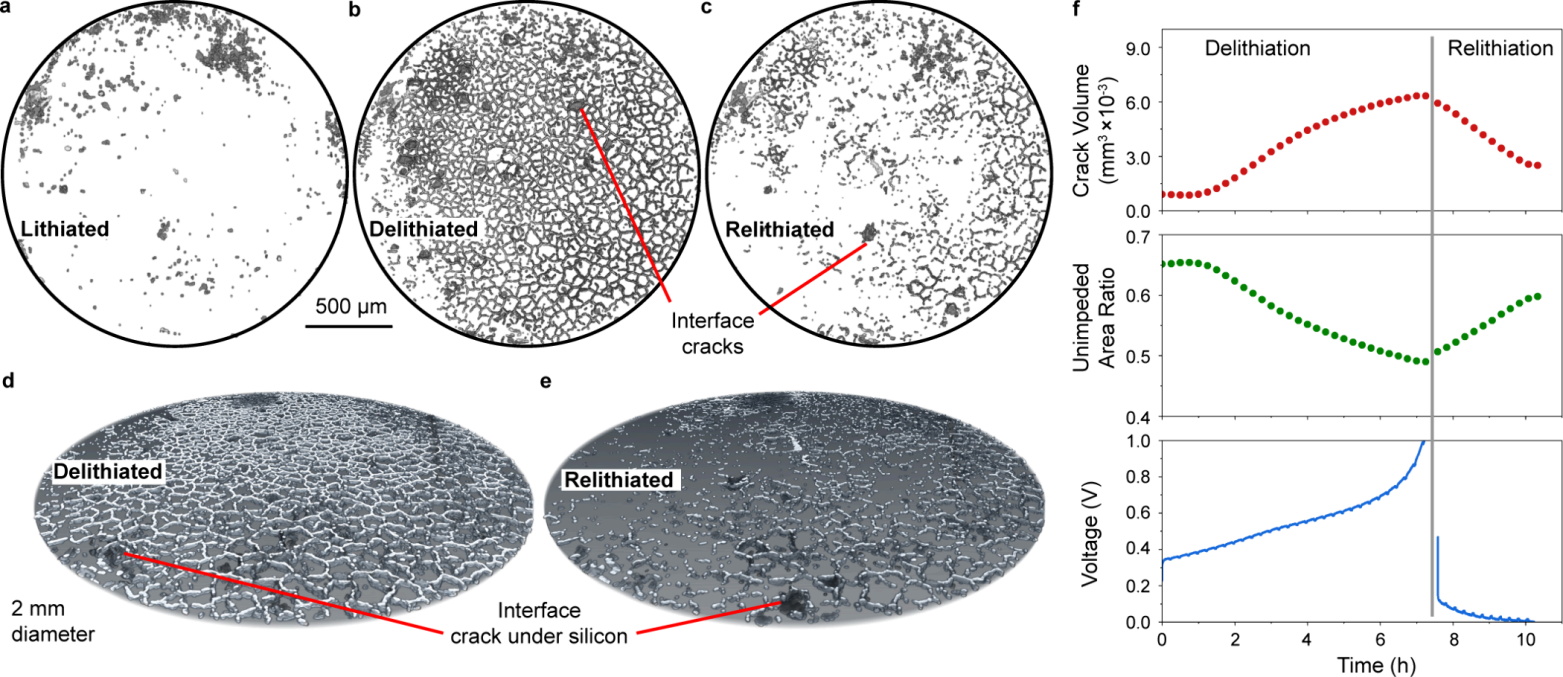
Quantifying fracture processes with image segmentation. (a–c) Renderings of the segmented 3D crack network for the (a) lithiated, (b) delithiated, and (c) relithiated states of the *operando* XCT experiment viewed from the current collector interface. (d,e) Tilted views of the 3D crack network in the (d) delithiated and (e) relithiated states with the silicon volume overlaid in gray. (f) Plots of total crack volume (top), unimpeded area ratio (middle), and galvanostatic voltage curves (bottom) with time during delithiation and relithiation.

The images in Figure 2a-e and Supplementary Video 2 provide a top-down view of the segmented crack network as it evolves during delithiation and relithiation. Resolvable channel cracks begin forming across the entire electrode after ∼1-h of delithiation. As these cracks widen, additional channel cracks linking them begin to form, uniformly increasing the crack density across the electrode as delithiation progresses. Interfacial cracks form sporadically later in the delithiation process in some regions enclosed by channel cracks. This suggests that local chemo-mechanical interactions between neighboring Li_*x*_Si domains, rather than bulk stress evolution across the entire electrode, contribute to interfacial fracture during delithiation. During relithiation, almost all the interfacial cracks vanish, and this occurs before the channel cracks surrounding them disappear. As the electrode is further relithiated, many channel cracks are closed, but some persist because the relithiation was only partial (Figure 1b).

The limited relithiation capacity arose because the cutoff voltage was reached prematurely compared to larger-diameter anvil cells (Figure S1). The smaller diameter of the tomography cell (2 mm) as compared to typical laboratory anvil cells (∼10 mm) results in a greater fraction of the SSE and electrode that are influenced by interactions with the cells walls. We quantified the porosity within the SSE pellet via image segmentation and found greater porosity near the cell edges (Figure S3), which would increase impedance near the cell edges. Such edge effects are expected to play a greater role in cells with smaller areas. We thus believe that the lower reversibility of the tomography cell was largely due to cell edge effects, but that this does not affect the local and global fracture mechanisms we identify and investigate herein.

The dynamics of fracture described above were quantified by tracking the segmented crack volume and the “unimpeded area ratio” with time (Figure 2f). The unimpeded area ratio is defined as the fractional electrode area through which there is a continuous visible pathway through electrode material (i.e., there is no void voxel in each column of voxels between the current collector and the SSE). The total crack volume and unimpeded area ratio follow inverse S-shaped trends (Figure 2f). There is a ∼1-h period at the beginning of delithiation before the channel cracks appear (Supplementary Videos 1 and 2). The total crack volume then starts increasing rapidly as the channel crack network opens, resulting in a decrease in unimpeded area. This increase in crack volume continues as interfacial cracks start forming, but the rate of crack volume growth slows in the second half of delithiation as the existing cracks widen without forming any new cracks. The opposite trend is observed during relithiation, as the lithiation of silicon first near the LPSC interface causes the cracks to close near this interface. Some crack volume remains after relithiation (Figure 2c) due the partial reaction of the electrode, as discussed above. Figure 2c shows that some of the remaining crack volume is in the form of channel cracks, especially near the edges of the electrode, again suggesting spatially variable lithiation due to edge effects.

To shed light on the localized dynamics of channel-type and interfacial fracture, Figure 3 shows time series of different cross-sectional image slices during delithiation and relithiation (Supplementary Videos 3–5). We note that the relithiation portion is considerably shorter than the delithiation portion due to edge effects in the smaller cells, as discussed in the previous section. Figure 3a shows a local region that features channel cracking without substantial interfacial cracking. Some small voids were observed in the lithiated state before delithiation (top image of Figure 3a), though these are mostly unresolvable with image segmentation due to their small size compared to the larger cracks after delithiation. As delithiation progresses, the vertical channel cracks initially appear extended through the full electrode thickness and grow laterally as the Li_*x*_Si shrinks, becoming the widest at the end of delithiation. The vast majority of vertical channel cracks across the electrode become visible around the same time. Although the vertical cracks are already extended through the electrode thickness once they become resolvable, they must initiate and propagate through the electrode over time scales faster than the experimental temporal resolution (15 min). The LPSC interface also moves upward to accommodate the shrinking Li_*x*_Si as the opposing lithium counter electrode expands. During relithiation, the vertical cracks close near the LPSC interface as lithiation causes swelling near this interface.

**Figure 3:**
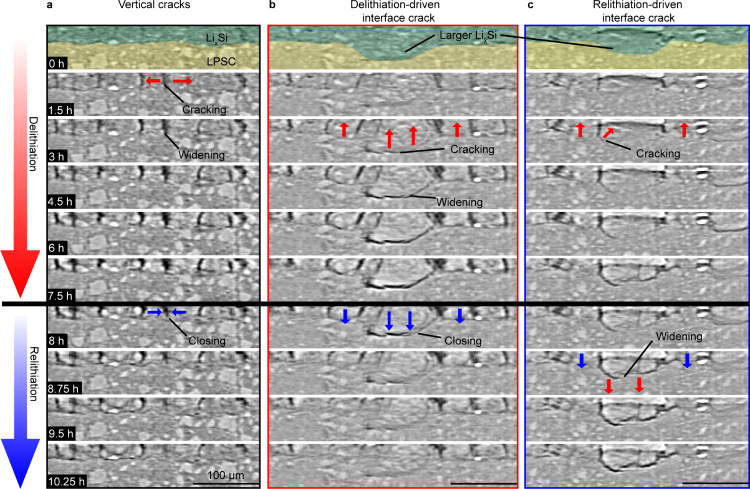
Time-series cross-sectional XCT image slices from different locations at the silicon/LPSC interface showing the delithiation and relithiation processes. (a) Images showing the formation and partial closure of the vertical channel-crack network. (b) Images showing delithiation-driven interfacial fracture caused by neighboring particle interactions. (c) Images showing relithiation-driven interfacial fracture again caused by neighboring particle interactions. The false-color overlays in the top row highlight the Li_*x*_Si and LPSC regions.

Figure 3b shows how delithiation-driven interfacial fracture typically occurs. In this region, there is a thicker Li_*x*_Si domain in the center of the image that extends ∼20 μm deeper into the LPSC than the neighboring Li_*x*_Si. As the Li_*x*_Si electrode is delithiated, this large domain shrinks preferentially, exhibiting a greater linear dimensional change because of its larger size (see SI Section S3). This results in severe interfacial delamination at the interface between the large domain and the LPSC as the domain shrinks, but less interfacial fracture for the surrounding thinner Li_*x*_Si domains. During relithiation, the crack closes within the first two image frames due to lithiation and swelling, indicating that this domain still retains sufficient contact to the interface or with neighboring domains to be electrochemically or chemically lithiated. Thus, the capacity associated with this material was not lost. Interfacial cracks were found to cover 1.5% of the total electrode area after delithiation (calculated from Figure 2b). Other *ex situ* SEM imaging studies have reported interfacial fracture across wide areas of silicon electrodes,^28^ which may artificially arise from delamination during sample preparation due to the need to remove the materials from the stack pressure in the cell. In contrast, our *operando* results show that delithiation-driven interfacial fracture occurs only under thicker silicon domains (Figure S4 shows other examples), indicating that divergent dimensional changes among neighboring domains of different thickness can intrinsically drive interfacial contact loss, a phenomenon unique to SSBs. This finding indicates that silicon electrodes with uniform thickness will likely provide enhanced interfacial contact retention and better performance, and future investigations of sputtered Si films with uniform thickness, as well as methods to improve the thickness uniformity of slurry-cast electrodes, would be beneficial.

Figure 3c shows the process by which a Si domain undergoes interfacial fracture upon relithiation. This region of the interface features a relatively large Li_*x*_Si domain as in Figure 3b, and there is evidence of minor fracture and contact loss at the LPSC interface upon delithiation as before. Due to a lack of transport pathways at the beginning of relithiation, this domain cannot swell as quickly as the surrounding silicon regions. The neighboring silicon expands and causes the LPSC interface to move downward before the larger domain can expand appreciably, resulting in growth of the interfacial crack and full separation of the large domain from the LPSC during relithiation. The large silicon domain is thus prevented from participating in electrochemical cycling due to delamination and represents “dead silicon.” This effect was observed for several other Li_*x*_Si domains and, similarly to the local dynamics in Figure 3b, the lithiation-driven interfacial fracture in Figure 3c only occurred at locally thicker silicon domains (Figure S5). Very few examples of this mechanism were observed, comprising only 0.85% of the total electrode area (calculated from Figure 2c). These findings indicate that relithiation-driven interfacial fracture occurs due to reaction competition from neighboring Li_*x*_Si domains and highlights the importance of understanding dynamic relationships between evolving domains at solid–solid electrochemical interfaces.

The data in Figures 1-3 are from a half cell, and to verify that these observed fracture mechanisms are not strongly affected by the choice of counter electrode with different volume change characteristics, we performed *ex situ* XCT imaging on a silicon-anode full cell with an NMC-622/LPSC composite cathode (Figure S6). Vertical channel cracking is readily apparent in the delithiated state, creating individual delithiated silicon domains as in the half cell. The average diameter of the delithiated silicon domains after delithiation in the full cell is 50.6 ± 7.6 μm, which is larger than the average diameter of the domains in the half cell in Figure 1-3 (29.5 ± 5.4 μm). This size difference may be influenced by the different Coulombic efficiencies of these experiments (60.7% for the half cell compared to 45.8% for the full cell) resulting in different degrees of delithiation and shrinkage of the Li_*x*_Si domains. Still, several delithiation-driven interfacial cracks are observed under larger silicon domains in the full cell (Figure S6), similar to the half cell. In both the half and full cell data sets, we stress that the formation of smaller interfacial cracks that are not resolvable by XCT is possible, but the interfacial cracks resolved herein in both cells appear to form via similar mechanisms. While extended cycling data were not obtained, it is possible that these interfacial cracks would worsen over cycling due to accumulated degradation at the interface. This would result in an increasing number of silicon domains becoming isolated with cycling. Overall, these data provide firm evidence that the evolution of interfacial contact in silicon anodes depends strongly on local inhomogeneities, thickness variation, and competition among neighboring domains rather than being influenced by the counter electrode.

To further quantify the factors governing interfacial fracture, the differences in thickness between Li_*x*_Si domains and their surroundings were averaged for all domains that underwent delithiation-driven interfacial fracture. In the *operando* half cell experiment, the average initial thickness difference between the 10 Li_*x*_Si domains that underwent delithiation-induced interfacial fracture and surrounding domains was 24.2 μm ± 8.6 μm, with 13.3 μm being the smallest observed thickness difference leading to fracture. After delithiation, the average thickness difference decreased to 20.7 μm ± 6.4 μm. In the *ex situ* full cell experiment, the average thickness difference measured for the six observed fractured domains after delithiation was 22.6 μm ± 14.8 μm (Figure S6), which agrees with the half cell data. Importantly, in both cells, all domains with this range of thickness variation showed interfacial fracture behavior. These quantified values suggest that variations in thickness between neighboring silicon domains that are on the order of 15–25 μm will result in severe degradation of the Li_*x*_Si/SSE interface. This also suggests that the maximum silicon particle size in slurry-cast electrodes must be kept below these values to prevent protrusion of individual silicon particles.

To consider the chemo-mechanical evolution of silicon electrodes in SSBs in the context of electrochemical cycling, *in situ* potentiostatic electrochemical impedance spectroscopy (EIS) was performed on a 1 cm-diameter anvil-type silicon half cell with Li_6_PS_5_Cl (LPSC) SSE and a lithium counter electrode. The working electrode consisted of 99.0 wt % silicon microparticles slurry-cast on copper foil with an areal loading of 1.75 mg Si cm^–2^ (6.25 mAh cm^–2^). Figure 4a shows the voltage profile of the cell during the first two cycles; the voltage relaxations at intervals of 0.4 mAh cm^–2^ correspond to rest periods when EIS was performed. The cell shows increased reversibility compared to the smaller tomography cells due to a reduced influence of edge effects, as already described.

**Figure 4:**
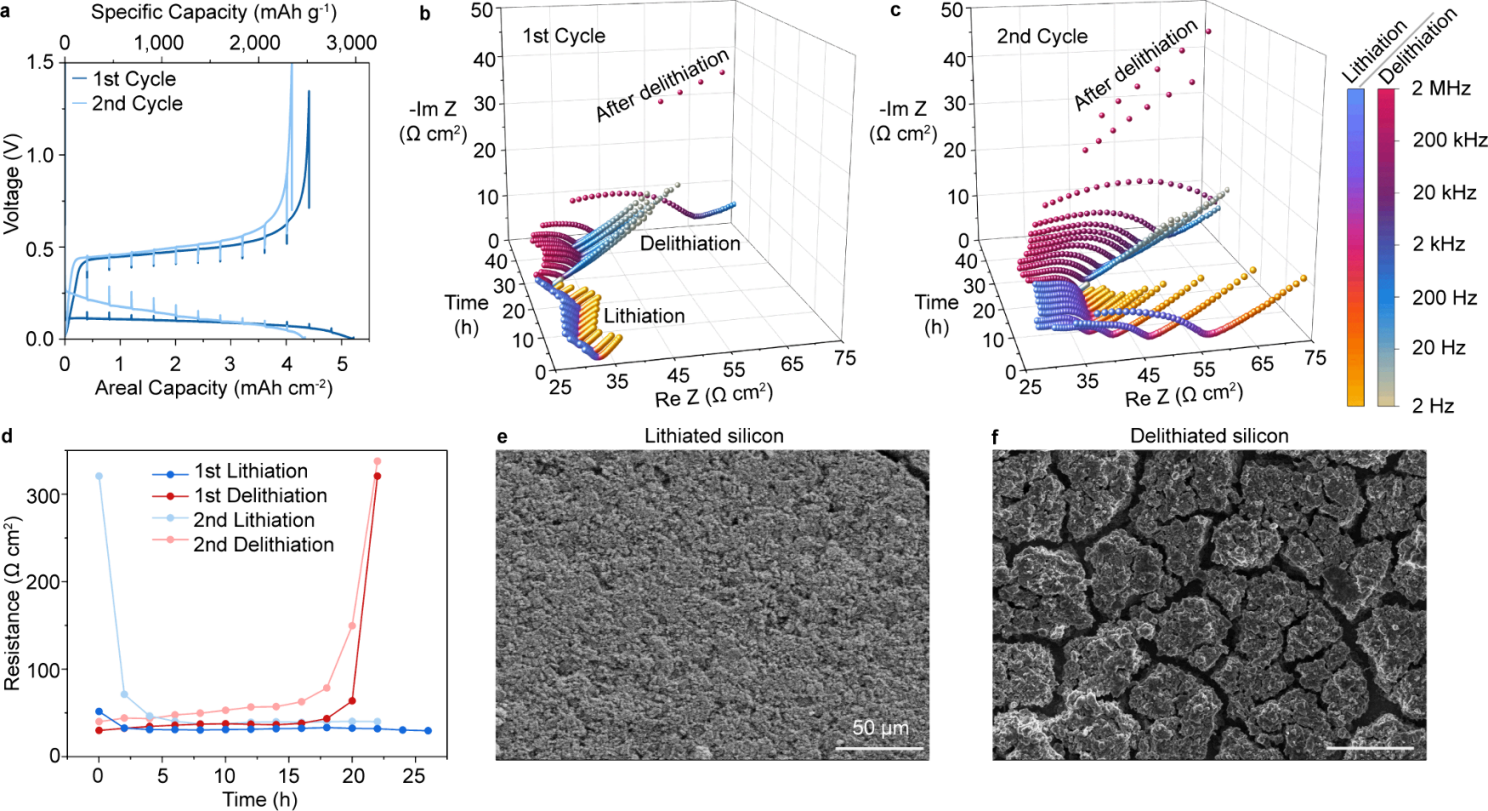
*In situ* EIS of a silicon anode solid-state battery half cell with corresponding *ex situ* SEM images. (a) Voltage curves from an anvil-type silicon half cell under galvanostatic testing during which EIS spectra were collected at intervals of 0.4 mAh cm^–2^. The cell was cycled twice at 25 °C under 10 MPa stack pressure and a current density of 0.2 mA cm^–2^. (b,c) Nyquist plots from the two cycles shown in (a) during (b) the first and (c) the second cycles. (d) Extracted total area-specific resistance plotted against time for each half cycle of the *in situ* EIS experiment. (e,f) *Ex situ* SEM images of (e) lithiated and (f) delithiated silicon electrodes removed from cells viewed from the interface that formerly contacted the current collector.

Figure 4b-c displays the impedance spectra collected during the first two cycles of this cell. Note that control experiments on Li/Li symmetric cells with similar areal capacities showed only very minor shifts of the spectra with no change of the spectral shape, indicating that the Li counter electrode did not significantly contribute to impedance evolution under these conditions (Figure S7). The Nyquist plot in Figure 4b reveals a small drop of impedance during the first lithiation when the silicon electrode expands. The spectral shape during the first lithiation consists of a partial semicircle at high frequencies and a short tail at lower frequencies, with an approximate semicircle width of ∼32 Ω cm^2^ (Figure 4b). There is then a continuous increase of impedance during the first delithiation (Figure 4b), with the inflection point at ∼2 kHz in the spectra increasing almost 10-fold from 30 Ω cm^2^ initially to 321 Ω cm^2^ after delithiation. The first-cycle Coulombic efficiency was 84.6%.

The *ex situ* SEM images in Figure 4e-f show lithiated and delithiated silicon electrodes cycled in half cells. The initially particulate material (Figure S2) became pulverized and densified after lithiation (Figure 4e), followed by extensive fracture after delithiation with cracks aligned perpendicularly to the SSE interface (Figure 4f).^10^ The average diameter of the silicon domains in Figure 4f found by measuring in two perpendicular directions is 30.8 μm ± 5.3 μm, which is similar to that measured in the *operando* XCT cell (29.5 μm ± 5.4 μm). To further verify that the choice of counter electrode does not affect the observed microstructural evolution, *ex situ* SEM images of silicon anodes cycled in full cells vs LiNi_0.6_Co_0.2_Mn_0.2_O_2_ (NMC622) were also collected (Figure S8),^48−50^ and both sets of data show similar microstructures.

The second cycle (Figure 4c) shows a similar EIS trend as the first cycle, with a decrease of impedance during lithiation and an increase during delithiation. However, the widths of the spectra remain larger throughout the second cycle (Figure S9 shows demagnified spectra). *Ex situ* SEM images after the second cycle are provided in Figure S10. The spectra for the second cycle also feature a slightly different shape of the high frequency region compared to the first cycle, with an additional large semicircular feature between 200 kHz and 2 kHz.

Recent studies have shown that evolving interfacial contact complicates the analysis of EIS data in SSBs, potentially making linear equivalent circuit models inaccurate.^51−53^ To analyze the EIS spectra, we nevertheless use a simple equivalent circuit to extract the total resistance corresponding to the width of all semicircular features (Figure 4d; see Figure S11 for the equivalent circuits). Figure 4d shows that the overall resistance of the cells increases significantly near the end of delithiation, which is likely due to both interfacial and bulk electrode evolution leading to contact loss and current constriction, as detailed previously. Moreover, the resistance during the second delithiation cycle is higher than the first.

The electrochemical effects of crack formation are apparent in the context of these *in situ* EIS data in Figure 4b-d, although these measurements were on different cells and thus cannot be directly correlated. The vertical channel crack network and the interfacial cracks are likely the major contributors to the increasing impedance observed during delithiation. While most silicon domains within enclosed channel cracks can still participate as active material during delithiation, current constriction occurs at the edges of these regions of lost contact. Current constriction is known to exacerbate impedance increases because of locally high current densities.^51,52^ The relithiation process involves rapid crack closure near the LPSC interface, which likely reduces current constriction effects and correlates to the fast decrease in interfacial impedance visible in Figure 1c during second cycle lithiation.

To further investigate the links between fracture and electrochemical cycling, we developed a diffusion-deformation-damage continuum model which is solved using finite elements. A diffusion-deformation model^46^ is augmented to capture crack formation via a phase-field damage framework^54−56^ (see the SI and Figures S15 and S16 for model derivation). The framework captures the coupled species diffusion and concurrent elastic-plastic deformation and fracture of the silicon anode due to the large volumetric changes.^47,56−58^ The phase-field damage approach has the unique benefit that crack paths are not predefined and can evolve based on local stress state. Inherent microstructural heterogeneities are numerically modeled by prescribing a spatially varying uniform distribution of the silicon fracture toughness.

Figure 5a shows a representative two-dimensional plane-strain Li_*x*_Si/LPSC model in which the Li_*x*_Si is of uniform thickness. The Li_*x*_Si simulation domain (blue region in Figure 5a) is 150 μm by 10 μm and is attached to a 300 μm thick LPSC simulation domain (gray region in Figure 5a). The Li_*x*_Si domain is modeled through the coupled deformation-diffusion-damage continuum theory, while the LPSC electrolyte is treated as a purely linear-elastic material (see SI for details). The simulation thus mimics the experimental results shown in Figure 3a. Figure 5a illustrates the evolution of damage contours (i.e., fracture) across this Li_*x*_Si film during the first delithiation half-cycle (also see Supplementary Video 6). Note that damage *d* is defined to range from zero to one with *d* = 0 denoting the pristine undegraded material and *d* = 1 denoting the fully fractured solid (SI Section S4). Before the delithiation step, the initial lithiation step was also simulated, resulting in no observed damage since the film remains under compression. During delithiation, significant tensile stresses develop, leading to the formation and growth of multiple vertical channel cracks in qualitative agreement with the experiments. Consistent with the experimental observations, the simulated cracks propagate across the depth of the silicon film with an average spacing of ∼30 μm (see SI Section S5 and Figure S17). Figure S18 includes the corresponding contours of normalized state of concentration (c̅) and horizontal stress, (*T*_*xx*_). This agreement between simulation and experiment demonstrates that the modeling framework can be used to quantify aspects of stress and damage evolution.

**Figure 5:**
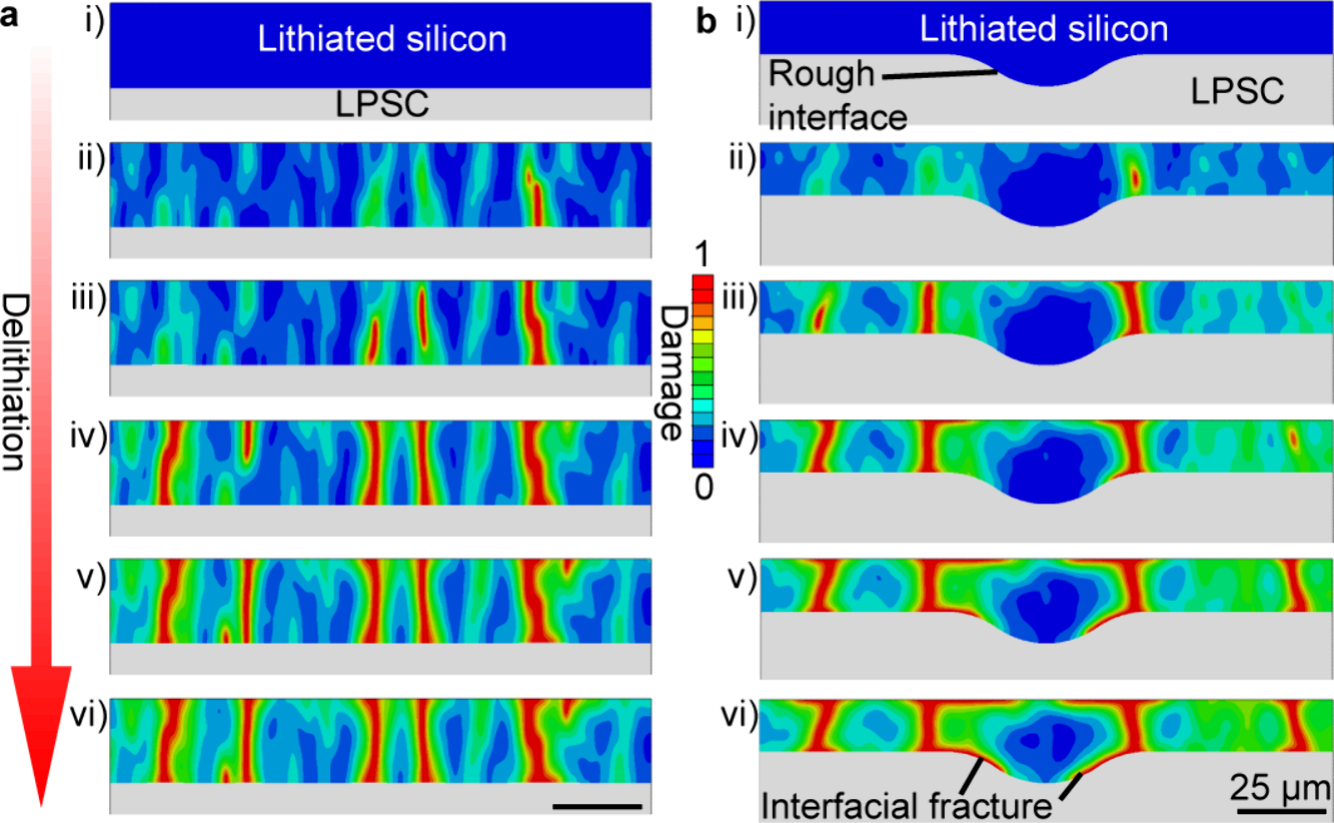
Phase-field modeling of the delithiation of Li_*x*_Si electrodes in contact with LPSC SSE. Contours of damage evolution in a Li_*x*_Si/LPSC simulation domain with Li_*x*_Si electrodes of (a) uniform thickness, with the initial Li_*x*_Si simulation domain being 150 μm by 10 μm and attached to a 300 μm thick LPSC domain, and (b) nonuniform thickness. The LPSC electrolyte is treated as a purely linear-elastic material (see SI).

Figure 5b shows another simulation with the Li_*x*_Si of nonuniform thickness, as experimentally observed in Figure 3b. During delithiation, vertical cracks form and subsequently grow underneath the thicker region at the Li_*x*_Si/LPSC interface. The simulated results are in qualitative agreement with the observed crack patterns shown in Figure 3b, including both the delamination of the interface and the concentration of vertical cracks away from the imperfection. The interfacial delamination is driven by the exacerbated vertical shrinkage of the thicker portion of the silicon electrode, leading to the presence of tensile stresses under the imperfection.

Further simulations showed that the initial stress state within the thick Li_*x*_Si domain influences the extent of interfacial fracture. Delamination during delithiation is concentrated beneath the imperfection when there is little stress built up during lithiation (Figure S19). In contrast, if compressive residual stresses are present in the Li_*x*_Si region after lithiation, interfacial fracture is expected away from the imperfection. This finding suggests that much of the stress associated with lithiation in the experiments is accommodated through the merging of the silicon particles and associated plastic deformation, resulting in little or no accumulation of stresses in the film at the end of the first lithiation cycle.

The simulation results further show that microstructural defects, introduced here though spatial variation of fracture toughness, do not govern the global crack pattern. Rather, as has been discussed in relation to fragmentation of silicon films, the spacing of the vertical cracks is primarily dictated by geometry (e.g., film thickness) and material properties (e.g., yield stress and toughness).^6,59,60^ To illustrate this, we performed simulations on flat Li_*x*_Si/LPSC interfaces with two distinct distributions of fracture toughness. Figure S17 illustrates the spatial distributions of fracture roughness and the corresponding simulated crack patterns. Irrespective of the variations in microstructural heterogeneity, vertical cracks at similar average spacing consistently propagate across the three silicon films. Additional simulations show that the crack spacing depends on film thickness,^59,60^ with simulations of thinner 6 μm thick films forming cracks with smaller spacing (20 μm) compared to the 10 μm films. Additionally, we performed sensitivity studies on lithium diffusivity, yield strength, and fracture toughness (Figures S20–S22).

Our *operando* experiments have revealed the formation of cracks governed by three mechanisms in silicon-anode SSBs: (1) vertical mud-type channel cracks that grow through the thickness of the silicon, (2) delithiation-driven interfacial fracture, and (3) relithiation-driven interfacial fracture, where (2) and (3) occur under relatively thick silicon domains. *In situ* EIS revealed that the increase of impedance during delithiation of silicon anode SSBs is likely correlated to the development of the large crack network throughout the silicon electrode. Modeling of the silicon/SSE system showed that interfacial fracture occurs due to magnified tensile stress under thicker domains, rather than global electro-chemo-mechanical phenomena. Likewise, regions of the silicon electrode that are relatively uniform in thickness are unlikely to experience interfacial fracture and will thus retain lower interfacial impedance, suggesting that methods for producing highly uniform electrodes (such as sputtered films or uniformly calendered pure and composite particulate electrodes) over large areas will enhance performance.

While imaging of successive cycles was not possible during this experiment due to synchrotron time constraints, future *operando* XCT experiments exploring different applied stack pressures over multiple cycles would reveal how the observed degradation mechanisms in this work evolve over extended cycling and enhance our understanding of these mechanisms as they apply to silicon SSBs operating under commercially relevant conditions. Furthermore, investigation of the effects of adhesion between silicon and various SSEs on interfacial fracture is needed. Future experiments to quantify the effects of different binders, SSEs, other additives, and stack pressures on the degradation mechanisms observed herein, particularly with higher-resolution imaging techniques, will aid in the optimization of silicon anodes for use in SSBs. Our work demonstrates that *operando* visualization of dynamics in emerging battery systems is critical for understanding new mechanisms that govern behavior, and it provides a step forward in the understanding and control of high-capacity alloy electrodes for SSBs.

## Supporting Information Available

Experimental details, materials, and methods, including photographs of experimental setup and phase-field modeling process, battery cycling data, additional X-ray CT image data and analysis, and SEM characterization (PDF)
Top-down XCT video of an image slice of the silicon electrode in the *operando* XCT experiment during delithiation (1–4 s) and relithiation (5–7 s) (MOV)
Top-down views of the segmented crack network in the silicon electrode in the same orientation as Video S1 during delithiation (1–4 s) and relithiation (5–7 s) (MOV)
Time-series of cross-sectional XCT image slices shown in Figure 3a (MOV)
Time-series of cross-sectional XCT image slices in Figure 3b (MOV)
Time-series of cross-sectional XCT image slices in Figure 3c (MOV)
Time-series of phase-field modeling simulations in Figure 5 (MOV)

## Supplementary Material

nz4c02800_si_001.pdf

nz4c02800_si_002.mov

nz4c02800_si_003.mov

nz4c02800_si_004.mov

nz4c02800_si_005.mov

nz4c02800_si_006.mov

nz4c02800_si_007.mov
