# Supplementary material for: Fracture Dynamics in Silicon Anode Solid-State Batteries
Source: ACS Energy Lett. 2024 Nov 26;9(12):6085–95. doi: 10.1021/acsenergylett.4c02800 (PMC11650773; doi:10.1021/acsenergylett.4c02800)
Supplement: Supplementary file 1 — nz4c02800_si_001.pdf [file nz4c02800_si_001.pdf]

SUPPORTING INFORMATION

Fracture Dynamics in Silicon Anode Solid-State Batteries

D. Lars Nelson[1], Stephanie E. Sandoval[1], Jaechan Pyo[2], Donald Bistri[2], Talia A. Thomas[3], Kelsey Anne Cavallaro[1], John A. Lewis[1], Abhinav S. Iyer[3], Pavel Shevchenko[4], Claudio V. Di Leo*[2], and Matthew T. McDowell*[1,3]

AFFILIATIONS

1. School of Materials Science and Engineering, Georgia Institute of Technology, Atlanta, GA, 30332, USA
2. Daniel Guggenheim School of Aerospace Engineering, Georgia Institute of Technology, Atlanta, GA, 30332, USA
3. George W. Woodruff School of Mechanical Engineering, Georgia Institute of Technology, Atlanta, GA, 30332, USA
4. Advanced Photon Source, Argonne National Laboratory, Lemont, IL, USA

Experimental Section

Silicon electrode preparation: Silicon electrodes were prepared with crystalline silicon powder (Sigma-Aldrich, 325 mesh), n-methyl-2-pyrrolidone (NMP) solvent (Sigma-Aldrich), and polyvinylidene fluoride (PVDF) binder (Sigma-Aldrich) using 1.0% PVDF and 99.0% silicon by mass. Slurry casting was performed with a tape caster (MTI) and doctor blade. Slurries were dried overnight in air at 50 °C. The areal loadings were 1.75 mg Si cm^{-2} (6.25 mAh cm^{-2}) for the *in situ* EIS experiment and 2.0 mg Si cm^{-2} (7.15 mAh cm^{-2}) for the *operando* XCT experiment. Electrodes were punched out with a 3/8-in diameter hole punch for anvil cell assembly and a 2 mm diameter hammer punch for tomography cell assembly.

LNTO-coated NMC-622 composite cathode: Inside an argon-filled glovebox, 20 mg lithium acetate (Sigma-Aldrich) was combined with 4 mL anhydrous ethanol (Sigma-Aldrich), 36 µL niobium ethoxide (Sigma-Aldrich), and 60 µL tantalum butoxide (Sigma-Aldrich) and stirred at 250 RPM for 6 h or until all lithium acetate was dissolved. Single-crystal NMC-622 (MSE Supplies) was combined with the solution in a ratio of 2 g NMC / 1 mL solution and then sonicated for 1 h. Outside of the glovebox, the mixture was placed in a vacuum oven overnight at 70 °C until all solvent was evaporated. In a steel-lined crucible, the dried powder was heated to 300 °C over 30 min and held at this temperature for 10 h, and it was then heated to 450 °C over 15 min and held at this temperature for 1 min before cooling to room temperature. In an argon-filled glovebox, the LNTO-coated NMC-622 powder was combined with LPSC and carbon nanofibers (CNF) in a mass ratio of 70% NMC-622 / 27.5% LPSC / 2.5% CNFs and then ball milled three times for 15 min at 150 RPM with 5 min of rest between each cycle.

Solid-state anvil cell assembly: Solid-state anvil cells were assembled inside an argon-filled glovebox (MBraun, <4 ppm O_2) with silicon electrodes, commercial LPSC (MSE Supplies,

ultrafine particle size (~1 micron)), and either 70% LNTO-coated NMC-622 composite cathode (full cells) or lithium foil (MSE Supplies, half cells) in custom-made cell housings consisting of a PEEK cylinder with a 10 mm inner diameter and two titanium rods (just under 10 mm diameter) contained by two steel plates and 4 screws with hex nuts.[1] LPSC was first pressed in the PEEK cylinder at 100 MPa to keep the LPSC pellet in place. The silicon electrode was then added, and the stack was pressed at 325 MPa for 5 min, followed by a release period of 1 min. For full cells, enough 70% LNTO-coated NMC-622 composite cathode was added to the opposite side of the LPSC to achieve an N:P ratio of 1.2 before pressing to 325 MPa for 5 min. For half cells, 3/8-in diameter lithium foil was added to the opposite side of the LPSC after the 325 MPa pressing step, and then the stack was pressed to 25 MPa for 5 min. A stack pressure of 10 MPa was applied to the battery during testing. Cells were cycled inside the argon-filled glovebox.

Solid-state tomography cell assembly: Solid-state tomography cells were assembled inside an argon-filled glovebox with silicon electrodes, commercial LPSC, and either 70% LNTO-coated NMC-622 composite cathode (full cells) or lithium foil (half cells) in custom-made cell housings consisting of a PEEK housing with a 2 mm inner diameter and two titanium rods contained by two hex-head screws with O-rings between the head of the screw and the edges of the PEEK housing.[2,3] LPSC was first pressed in the PEEK housing to keep the LPSC pellet in place by applying moderate pressure by hand with a titanium rod. The silicon electrode was then added, and the stack was pressed to >300 MPa for 5 min, followed by a release period of 5 min. For full cells, enough 70% LNTO-coated NMC-622 composite cathode was added to achieve an N:P ratio of 1.2 to the opposite side of the LPSC before pressing to >300 MPa for 5 min. For half cells, 2 mm diameter lithium foil was added to the opposite side of the LPSC after the >300 MPa pressing step. A stack pressure of 10 MPa was applied to the battery during testing with the two hex-head screws. Cells

were sealed using the O-rings between the screws and the PEEK and were cycled outside of the argon-filled glovebox for XCT imaging.

Electrochemical characterization: All electrochemical measurements and tests were performed using a Bio-Logic SP-200 potentiostat. Potentiostatic EIS scans were collected over a frequency range of 2 MHz to 2 Hz with 10 steps per decade. Lab-scale anvil cells were cycled at a current density of 0.2 mA/cm^2 in an argon glove box, while tomography cells were cycled at 0.5 mA/cm^2. Half cells had a lower voltage cutoff of 0.0 V and an upper voltage cutoff of 1.5 V for lab-scale cells and 1.0 V for the tomography cells. Full cells were cycled between 2.5 V and 4.0 V.

Scanning electron microscopy: SEM was performed on a Zeiss Ultra60 FE-SEM using an accelerating voltage of 8 kV and scanning speed of 6 during imaging.

X-ray computed microtomography: XCT was performed at Argonne National Laboratory's Advanced Photon Source (APS) on Beamline 2-BM. Monochromatic X-rays with an energy of 25.5 keV were used to image cells with a voxel size of 1.4 µm. 1500 projections, each with an exposure time of 400 ms, were collected over an angle of 180° with an Oryx 5.0 MP Mono 10GigE detector and a 2x magnification lens. 2 mm diameter tomography cells were made in a custom solid-state cell housing (Figure 2a) that allowed for stack pressure to be maintained throughout cycling while keeping the battery materials sealed in argon.

Digital image processing: Image reconstruction was performed at APS with the TomoPy toolbox for Python using the Gridrec method.[4] Image segmentation was performed in MATLAB 2023a by first converting all scans to 16-bit depth through a normalized scale and then applying a bilateral filter (Gaussian smoothing with edge preservation) to each image using a standard deviation of 3.0 (detailed in the SI and Figs. S12-S14). Intensity-based thresholding was applied to each image to

isolate the crack network. 3D reconstructions of the images and segmented volumes were created in ORS Dragonfly (non-commercial license version).

Phase-field modeling: Section S4 contains comprehensive information on the phase-field modeling framework.

Section S1 – Supporting electrochemical and imaging data

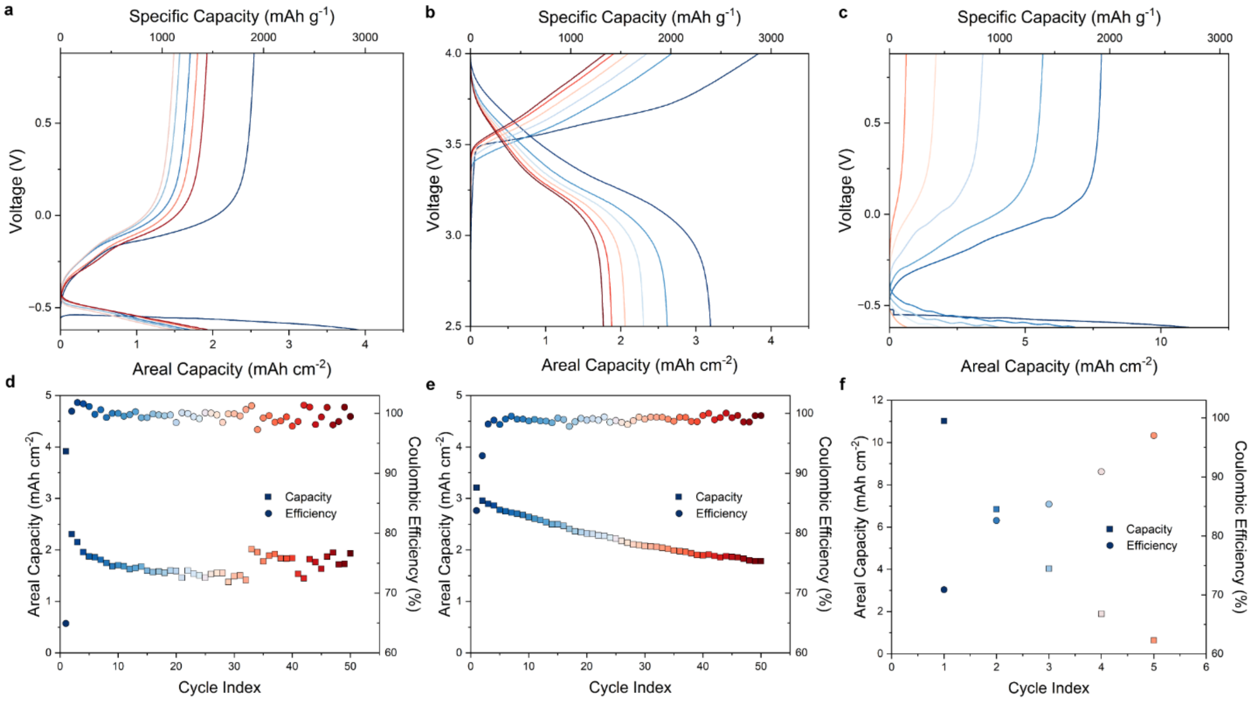

Figure S1. Long-term cycling data for Si-anode SSBs showing (a-c) voltage profiles and (d-f) areal capacity and Coulombic efficiency with cycling. All cells were operated under 0.5 mA cm^{-2} current density. (a, d) Cycling data from a Si/LPSC/LiIn anvil half cell, where the potential of Li/LiIn = 0.62 V vs. Li/Li^{+}. The stack pressure was 10 MPa. (b, e) Cycling data from a Si/LPSC/NMC-622 anvil full cell. The stack pressure was 30 MPa and the cathode areal loading was 4.5 mAh cm^{-2}. The N:P ratio was 1.2. (c, f) Cycling data from a Si/LPSC/LiIn tomography half cell. The stack pressure was 10 MPa. A higher areal capacity was used in this cell to replicate the higher capacities used in the XCT experiments.

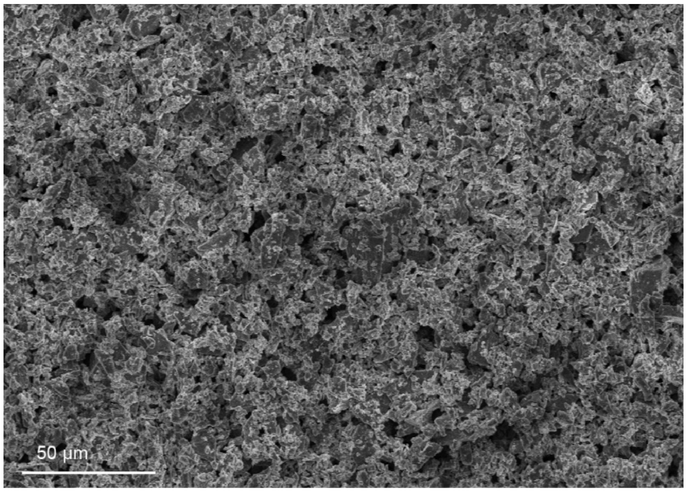

Figure S2. SEM image of a non-pressed, pristine silicon electrode after slurry-casting which was used to estimate the average particle size of the 325-mesh silicon before its inclusion in a solid-state cell.

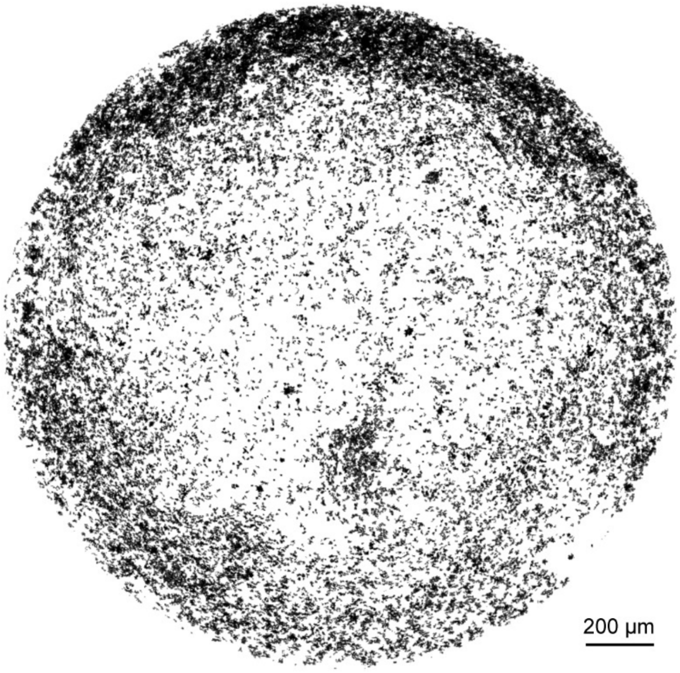

Figure S3. Top-down view of segmented porosity identified within the entire thickness of the SSE pellet used for the *operando* tomography experiment. There is increased pore density near the edges of the SSE that contact the PEEK wall.

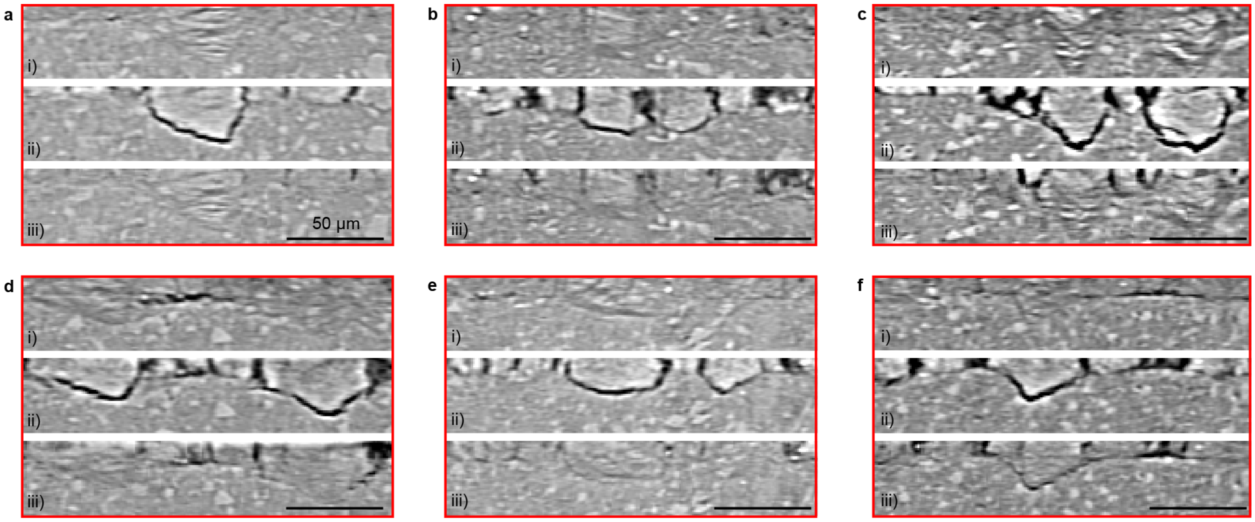

Figure S4. (a-f) Cross-sectional XCT images of all locations that display delithiation-driven interfacial fracture in the i) lithiated, ii) delithiated, and iii) relithiated states.

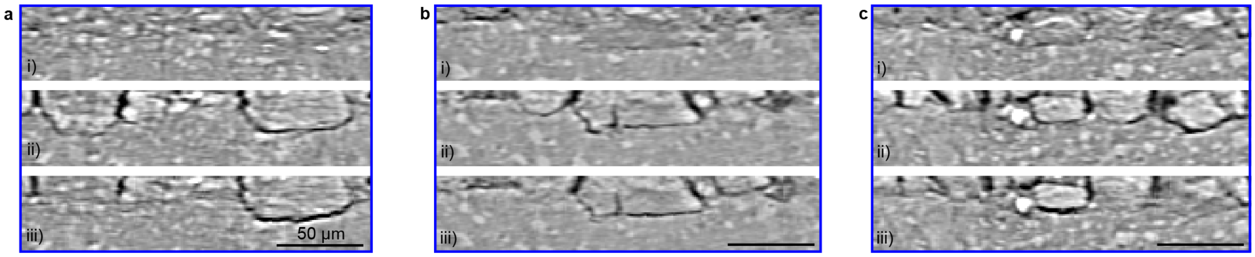

Figure S5. (a-c) Cross-sectional XCT images of all locations displaying relithiation-driven interfacial fracture causing the formation of "dead silicon" in the i) lithiated, ii) delithiated, and iii) relithiated states.

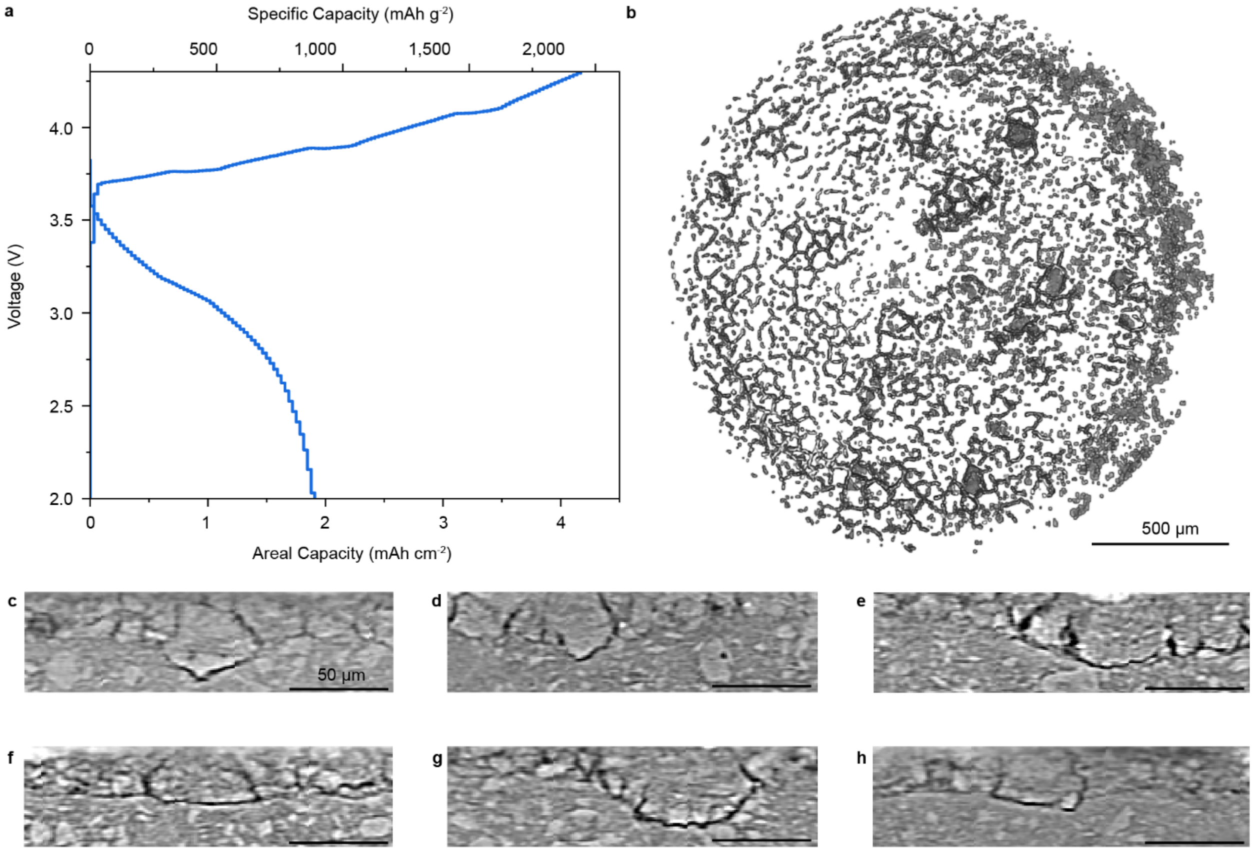

Figure S6. (a) Galvanostatic voltage profile of the silicon full cell cycled during the *ex situ* XCT experiment at 0.5 mA cm^{-2} current density, 10 MPa stack pressure, and 25 °C conditions. (b) Rendering of the segmented 3D crack network for the delithiated silicon anode observed from the interface that contacted the current collector. (c-h) Cross-sectional XCT images of all locations in the delithiated silicon anode that displayed delithiation-induced interfacial fracture.

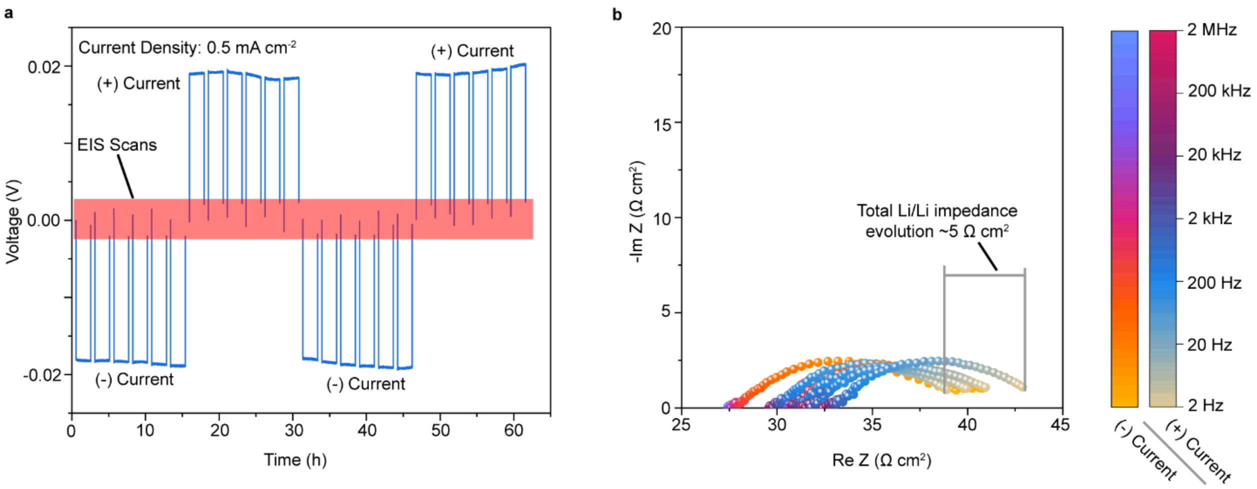

Figure S7. (a) Galvanostatic voltage profile of an anvil-type lithium foil symmetric cell with LPSC SSE. The cell was cycled with 6.0 mAh cm^{-2} areal capacity in each direction with *in situ* potentiostatic EIS scans performed every 1.0 mAh cm^{-2} (current density of 0.5 mA cm^{-2}). The red rectangle highlights the current-stop locations during cycling where EIS scans were performed. (b) *In situ* potentiostatic EIS spectra for the symmetric cell cycled in (a). The impedance evolves slightly with continued cycling (the furthest point increases from ~38.2 Ω cm^2 in the pristine state to ~43.0 Ω cm^2 after 24.0 mAh cm^{-2} total capacity has been cycled), but this amount is insignificant when compared to the increase in impedance during delithiation of the silicon half cell (Fig. 4b-c and Fig. S9).

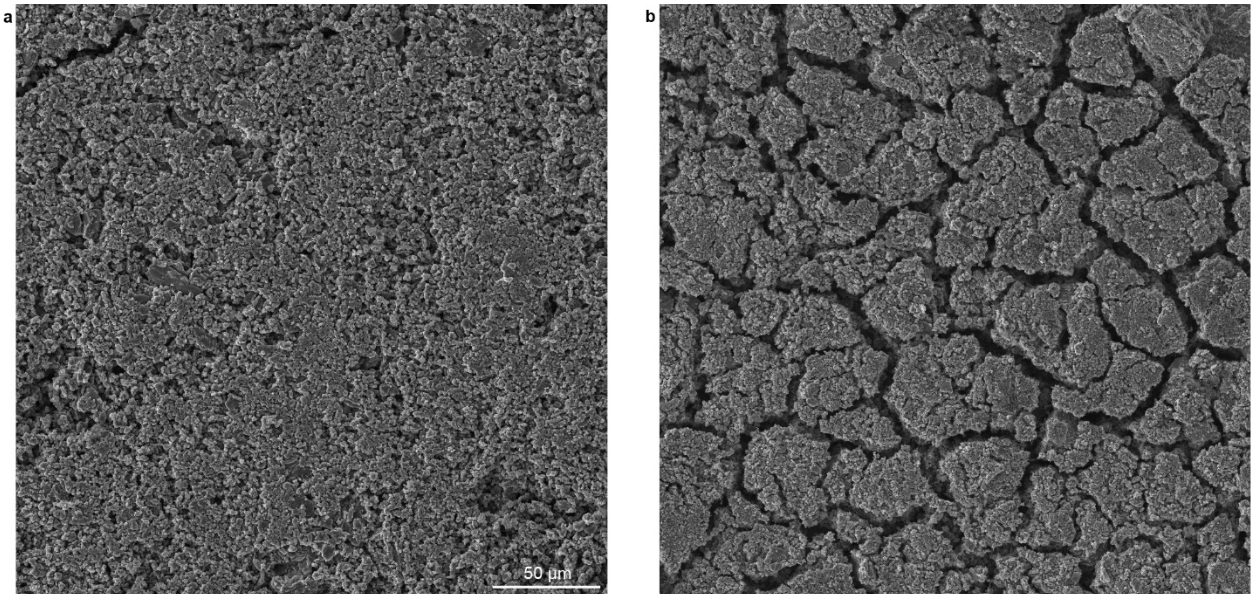

Figure S8. *Ex situ* SEM images of silicon electrodes cycled in full cells with NMC-622 / LPSC composite cathodes after (a) lithiation and (b) delithiation.

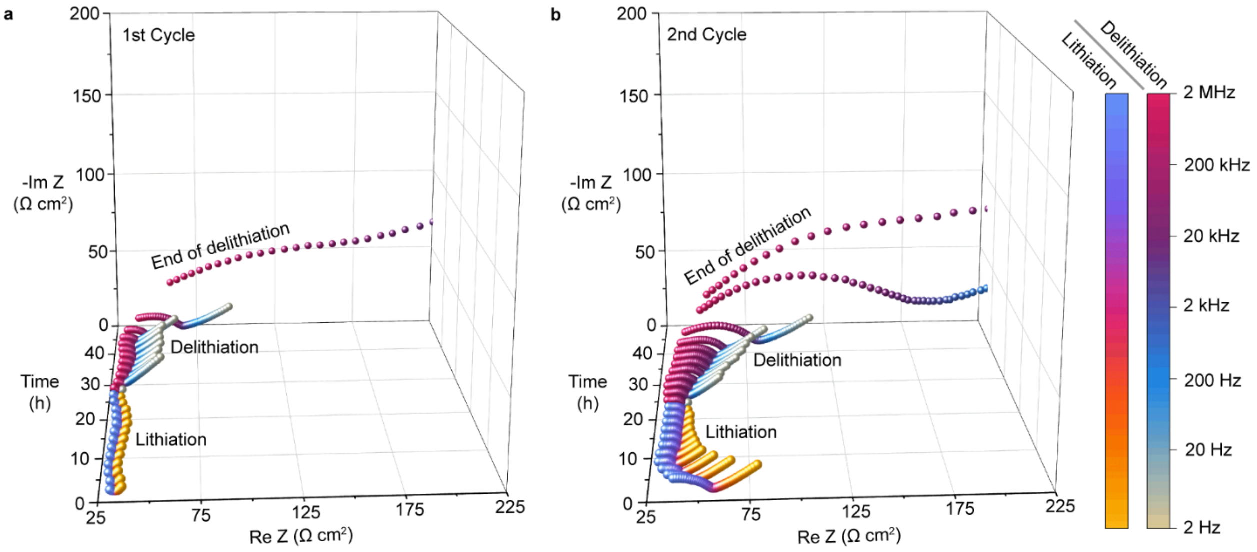

Figure S9. Demagnified views of the *in situ* potentiostatic EIS experiment shown in Fig. 4b-c for the (a) first cycle and (b) second cycle. This view highlights the end of delithiation stage where the largest impedance was recorded.

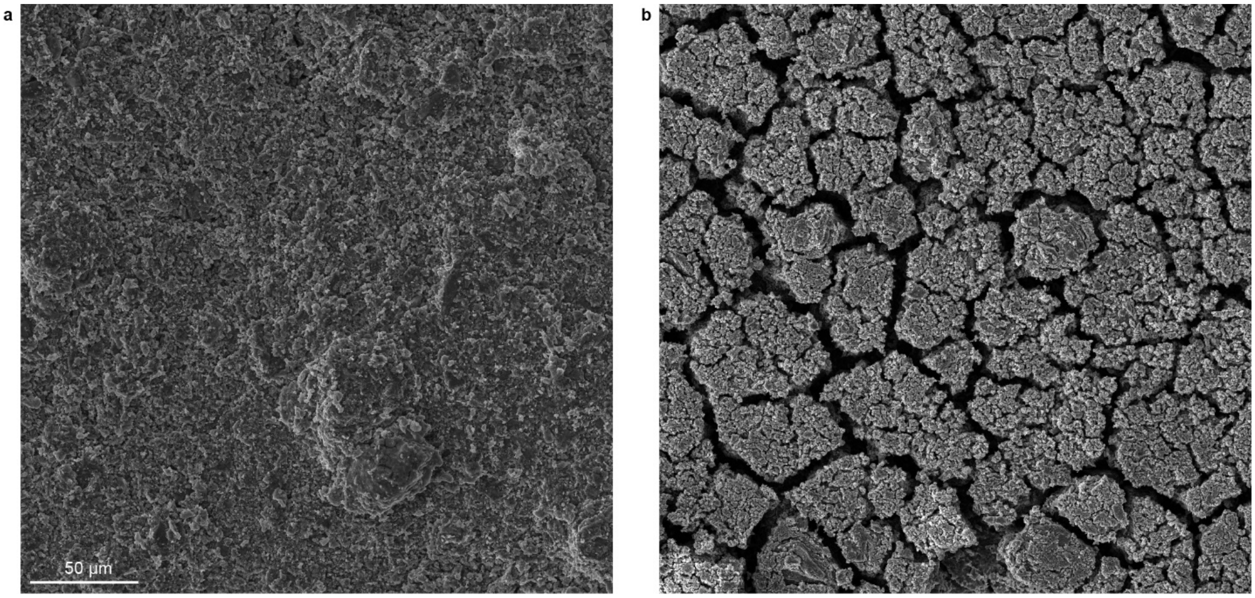

Figure S10. *Ex situ* SEM images of silicon electrodes cycled in half cells after (a) the second lithiation and (b) the second delithiation.

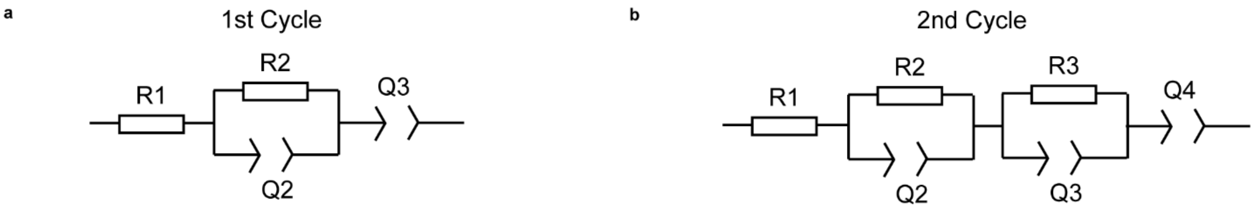

Figure S11. Linear equivalent circuit models used for the (a) 1st cycle and (b) 2nd cycle to obtain the total resistance plotted in Fig. 4d. Rectangles denoted by “RX” represent resistors, and the slanted breaks denoted by “QX” represent constant phase elements.

Section S2 – Image segmentation workflow

Image reconstruction was performed using the Gridrec method in TomoPy for Python. The reconstructed images had a voxel size of 1.4 µm. A sample image of the entire silicon half cell from the *operando* XCT experiment is shown in Fig. S12. The reconstructed images were in single-precision floating point format (bit depth of 32), so to improve computational speed they were first converted to 16-bit images. The bounds for rescaling each scan were set to the minimum and maximum intensity values found throughout the entirety of the 42 *operando* scans to ensure that the relative intensity of each scan was preserved. The preservation of the relative intensities of each scan was then verified by plotting the mean intensity of each scan to reveal a smooth, continuous evolution of intensity as cycling progresses and cracks develop and recede (Fig. S13). This indicates that the relative intensity of each scan was preserved during the conversion from 32 to 16-bit depth images.

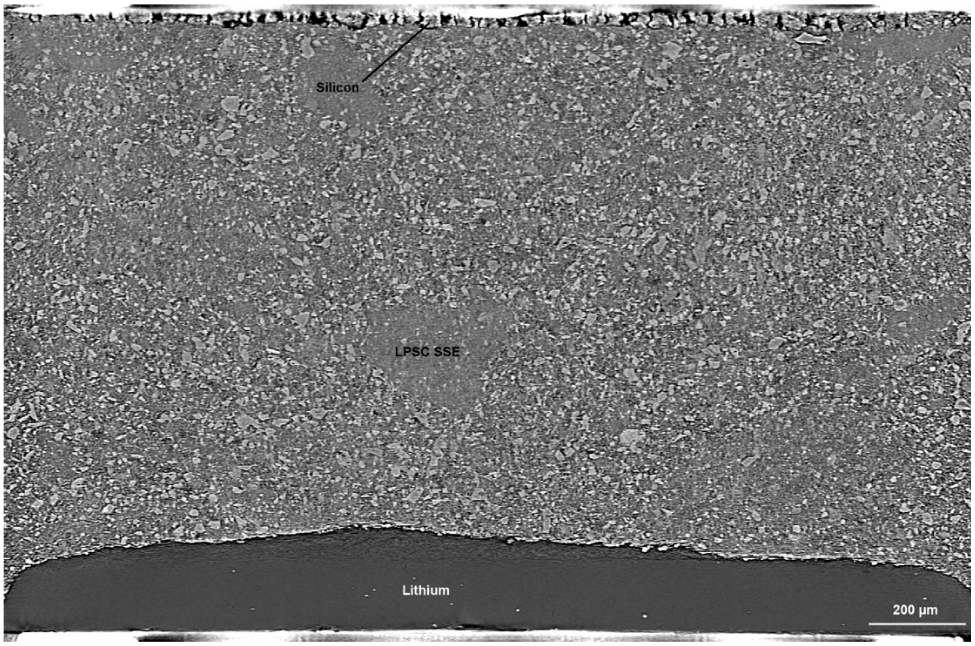

Figure S12. Sample cross-sectional XCT image of the entire delithiated silicon half cell used in the *operando* XCT experiment with labeled silicon, LPSC SSE, and lithium.

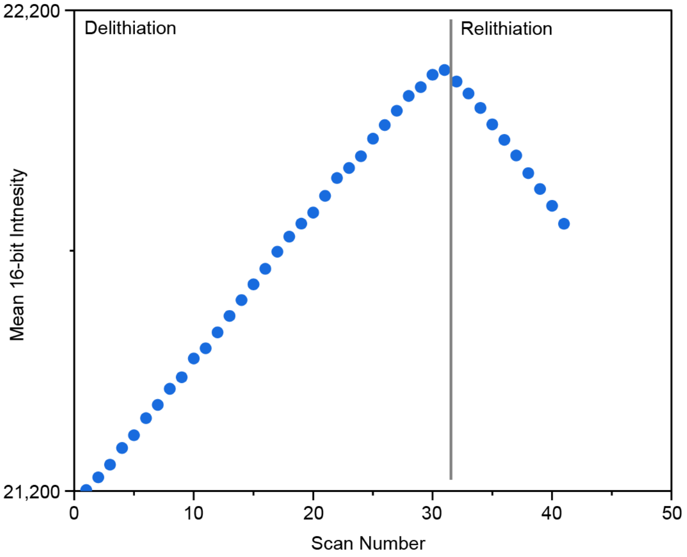

Figure S13. Mean 16-bit intensity of each *operando* scan from the silicon half cell experiment.

Following verification of the integrity of the image data, the silicon layer was cropped from each scan to isolate the area of interest and further reduce computational times (Fig. S14a). The top

voxel layer of the silicon that contacted the copper current collector was cropped out to avoid observing artifacts from the high X-ray absorbing metal.

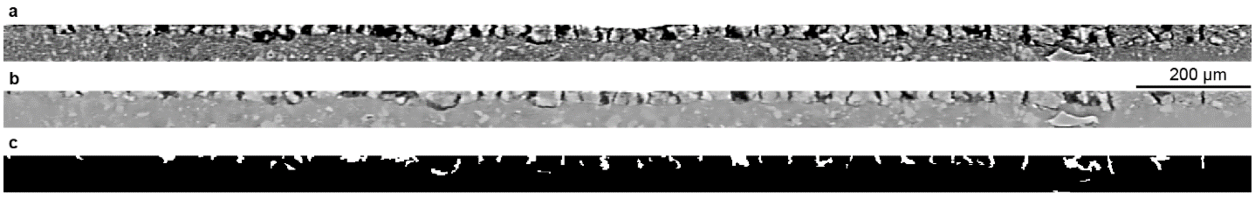

Figure S14. (a) Cropped silicon region from the cross-sectional XCT image in Fig. S12. (b) The same silicon cross-section after applying a bilateral Gaussian filter. (c) The results of intensity-based thresholding segmentation of the image in (b).

After cropping each scan to the same size in all three dimensions, a bilateral filter was applied to each scan (Fig. S14b). A bilateral filter applies a Gaussian blur to the image, but accounts for a user-specified standard deviation between pixels beyond which edges are preserved. Thus, similar intensity pixels (such as those in the different LPSC particles or within uncracked silicon domains) are smoothed together to remove texture effects while sections with drastically different intensities (such as the transition from a silicon domain to a crack) retain their sharp edges. The bilateral filter aided in isolating the silicon crack network from the silicon and LPSC while also removing small pores within the solid electrolyte. After filtering the images, an intensity-based threshold was applied using the provided MATLAB code in the supplementary materials to separate the crack network from the silicon and LPSC entirely (Fig. S14c).

Section S3 – Volume-dependent nonuniform thickness shrinkage during delithiation

Consider two separate fully-lithiated silicon domains, L (large) and S (small), where L has a volume twice that of S ($V_L = 2V_S$). For simplicity, assume that both domains are spherical. The radius of each domain can then be related to the volume of each domain by:

$$r_{L,0} = \left(\frac{3}{4\pi} V_L\right)^{1/3} \tag{1}$$

$$r_{S,0} = \left(\frac{3}{4\pi} V_S\right)^{1/3} \tag{2}$$

Using the relationship between the two volumes ($V_L = 2V_S$), the radius of the smaller domain can be related to the volume of the larger domain as such:

$$r_{S,0} = \left(\frac{3}{8\pi} V_L\right)^{1/3} \tag{3}$$

Because silicon can expand and contract by over 300% during lithiation and delithiation,[5,6] assume that the final volume of each domain after delithiation is one-third of its original volume. The delithiated radius of each domain is then given as:

$$r_{L,d} = \left(\frac{1}{4\pi} V_L\right)^{1/3} \tag{4}$$

$$r_{S,d} = \left(\frac{1}{8\pi} V_L\right)^{1/3} \tag{5}$$

The difference of each radius can then be found by subtracting the delithiated radius (4 and 5) from the original radius (1 and 3):

$$\Delta r_L = r_{L,0} - r_{L,d} = 0.2786 \left(\frac{V_L}{\pi}\right)^{1/3} \tag{6}$$

$$\Delta r_S = r_{S,0} - r_{S,d} = 0.2211 \left(\frac{V_L}{\pi}\right)^{1/3} \tag{7}$$

Thus, the relationship between the two new radii is given by:

$$\Delta r_S = 0.7936 \Delta r_L \tag{8}$$

In summary, for the same relative volume change during delithiation, the linear dimension (the radius) of each domain will change unequally, with the larger domain experiencing greater absolute shrinkage than the smaller domain. This effect is likely the cause of the delithiation-driven interfacial fracture observed in the *operando* XCT experiment.

Section S4 – Theoretical framework and numerical implementation

I. Summary of the theory

We present here, for completeness, a brief summary of the coupled diffusion-deformation-damage theory for modeling the behavior of silicon electrodes in our simulations. The theory closely follows the work of Di Leo *et al.*,[7] extended here to concurrently account for the mechanical fracture of silicon electrodes during lithiation/delithiation. To model the coupled species diffusion with large elastic-plastic deformations due to volumetric changes incurred by the diffusing species, the framework invokes a multiplicative decomposition of the deformation gradient ($\mathbf{F}$) into elastic ($\mathbf{F}^e$), plastic ($\mathbf{F}^p$), and swelling ($\mathbf{F}^s$) distortions,

$$\mathbf{F} = \mathbf{F}^e\mathbf{F}^p\mathbf{F}^s \tag{9}$$

Here, the distortion tensors, $\mathbf{F}^s$and $\mathbf{F}^p$, jointly model the kinematics associated with volumetric changes due to species diffusion, captured by $\mathbf{F}^s$, and the concurrent plastic deformations captured through $\mathbf{F}^p$. Any ensuing deformations of elastic nature are captured through the distortion tensor ($\mathbf{F}^e$).

Based on the isotropy of amorphous silicon (a-Si), the swelling distortion ($\mathbf{F}^s$) is taken to be spherical in form and linearly dependent on the state of lithium concentration through,

$$\mathbf{F}^s = (J^s)^{1/3}\mathbf{1}, \text{ with } J^s = 1 + \Omega\left(c_R - c_{R,0}\right) \tag{10}$$

with, Ω denoting a constant partial molar volume of the alloying lithium in the host, c_R the concentration of lithium per unit reference volume, and $c_{R,0}$, the initial concentration of lithium.

Consistent with our earlier work[7], the Mandel ($\mathbf{M}^{\mathrm{e}}$) and Cauchy ($\mathbf{T}$) stress tensors are then given by,

$$\mathbf{M}^{\mathrm{e}} = g(d)\left\{2G(\overline{c})\mathbf{E}^{e} + \left[K(\overline{c}) - \tfrac{2}{3}G(\overline{c})\right]\mathrm{tr}(\mathbf{E}^{e})\mathbf{1}\right\} \tag{11}$$

$$\mathbf{T} = g(d)J^{e^{-1}}\left\{2G(\overline{c})\mathbf{E}^{e}_{H} + \left[K(\overline{c}) - \tfrac{2}{3}G(\overline{c})\right]\mathrm{tr}(\mathbf{E}^{e}_{H})\mathbf{1}\right\} \tag{12}$$

with $G(\overline{c})$ and $K(\overline{c})$ denoting the concentration dependent shear and bulk modulus, respectively, while $\{\mathbf{E}^{e}, \mathbf{E}^{e}_{H}\}$ are the referential and spatial elastic logarithmic strain tensors respectively. To model the inability of fractured electrode regions to sustain tensile strains during delithiation, both stress measures are accordingly penalized, as is conventionally done in the literature,[8–10] by a monotonically increasing degradation damage function ($g(\mathrm{d})$) of the form,

$$g(\mathrm{d}) = (1 - \mathrm{d})^2 + \varepsilon \tag{13}$$

Here, $\mathrm{d}(\mathcal{X}, t) \in [0, 1]$ denotes a phase-field damage variable, which describes the state of fracture incurred on the silicon electrode, such that a state of $\mathrm{d} = 0$ denotes the pristine undegraded material, while $\mathrm{d} = 1$ denotes the fully fractured host. Additionally, we assume microstructural changes leading to fracture to be irreversible and require damage to grow monotonically such that, $\dot{\mathrm{d}} \geq 0$. Lastly, $\varepsilon \approx 0$ represents a small positive-valued constant introduced to prevent ill-conditioning of the model when $\mathrm{d} = 1$.

The evolution equation for damage is adopted from the works of Anand *et al.*[8] and Miehe *et al.*,[9] and takes the following form,

$$\zeta\dot{\mathrm{d}} = 2(1 - \mathrm{d})\mathcal{H} - \psi^{*}\mathrm{d} + \psi^{*}\ell^{2}\Delta\mathrm{d} \tag{14}$$

Here, $\zeta > 0$ denotes a small viscous regularization parameter introduced to impart stability to our numerical solution scheme. Consistent with the derivations in Anand *et al.*,[8] in the rate independent limit ($\zeta \rightarrow 0$), the energy dissipated per unit volume as damage, d increases from 0 to 1 evaluates to ψ^*. Naturally then, in our formulation, ψ^* represents an energy per unit volume dissipated during the fracture process. As detailed later, ψ^* can be related to the experimentally reported fracture energy of the material of choice ($\mathcal{G}_c$) through a characteristic length scale parameter (ℓ) which controls the diffuse fracture zone. Lastly, $\mathcal{H}$ represents a monotonically increasing history function which ensures that the damage irreversibility constraint (i.e. $\dot{\mathrm{d}} \geq 0$) holds. We take this function to be of the form,

$$\mathcal{H} = \max_{s \in [0,t]} [\, \langle\, \psi_R^e(\mathbf{E}^e, c) \; - \; \psi^*/2 \,\rangle\,] \tag{15}$$

where $\psi_R^e(\mathbf{E}^e, c)$ is the positive mechanical elastic energy, details of which can be found in our earlier work[7].

To complete the mechanical portion of the theory, plastic distortions are taken to evolve according to,

$$\dot{\mathbf{F}}^p \; = \; \dot{\bar{\varepsilon}}^p \left(\frac{3}{2} \frac{\mathbf{M}_0^e}{\bar{\sigma}} \right) \mathbf{F}^p \text{ with } \bar{\sigma} \; \stackrel{\text{def}}{=} \; \sqrt{\frac{3}{2}} |\mathbf{M}_0^e| \text{ and } J^p \; = \; \det(F^p) \; = \; 1. \tag{16}$$

Here, the rate of change of plastic distortion tensor ($\dot{\mathbf{F}}^p$) is taken to be codirectional with the deviatoric Mandel stress ($\mathbf{M}_0^e$) and proportional in magnitude to the equivalent plastic strain rate ($\dot{\bar{\varepsilon}}^p$). Furthermore, $\bar{\sigma}$ denotes an equivalent tensile stress.

We introduce a positive-valued concentration-dependent yield stress, $Y(\bar{c}) > 0$ and employ a no flow condition (i.e. $\dot{\bar{\varepsilon}}^p = 0$) of the form, $\bar{\sigma} \leq Y(\bar{c})$. During plastic flow, $\dot{\bar{\varepsilon}}^p > 0$, the equivalent tensile stress is taken to be equal to a rate-dependent flow strength,

$$\bar{\sigma} = Y(\bar{c}) + Y_* \left(\frac{\dot{\bar{\varepsilon}}^p}{\dot{\varepsilon}_0}\right)^{1/n} \text{ with } Y(\bar{c}) = Y_{sat} + (Y_0 - Y_{sat}) \exp\left(-\frac{\bar{c}}{\bar{c}_*}\right) \tag{17}$$

Here, $Y_* > 0$ denotes a positive-valued, stress-dimensioned constant, $\dot{\varepsilon}_0$ is a reference tensile plastic strain rate, and n is a measure of the strain rate sensitivity of the material. Additionally, $\{Y_0, Y_{sat}, \bar{c}_*\}$ represent three positive-valued material properties, which enable for producing an exponential softening response from an initial flow strength value, Y_0 to a lower saturation value, Y_{sat} with $\bar{c}_*$ controlling the rate of decay.

Species diffusion is governed by the standard mass balance equation, $\dot{c_R} = -J\text{div}(\mathbf{j})$ with $J = \det \mathbf{F}$, and the spatial flux, $\mathbf{j}$ of the intercalating lithium taken to depend on the spatial gradient of the chemical potential (μ) as follows,

$$j = -m(grad\, \mu) \text{ with } m = g(\mathrm{d}) \frac{D_0}{R\vartheta} c(1 - \bar{c}) \tag{18}$$

where,

$$\mu = \mu_0 + R\vartheta \ln\left(\gamma \frac{\bar{c}}{1-\bar{c}}\right) - g(\mathrm{d}) \frac{1}{3} \mathrm{tr}(\mathbf{M}^e) \tag{19}$$

Here, m denotes the concentration dependent species mobility, D_o is the species diffusivity, R is the gas constant, ϑ is the absolute temperature, and γ is an activity coefficient fitted as $R\vartheta \ln(\gamma) = \sum_{n=2}^{7} a_n \cdot n \cdot \bar{c}^{(n-1)}$ to open circuit potential data[7]. Additionally, to limit the ability of fractured electrode regions to sustain the transport of ionic species, we monotonically degrade the species mobility through the damage-dependent penalty function, $g(\mathrm{d})$ introduced in (13).

Regarding the LPSC electrolyte behavior, consistent with its brittle ceramic nature, a linear elastic isotropic material response is assumed. Accounting for this, the mechanical behavior of the solid electrolyte can be fully described provided the elastic properties $\{E_{LPSC},\ \nu_{LPSC}\}$ are known.

II. Material Properties

The electro-chemo-mechanical properties for our silicon anode – LPSC electrolyte system are listed in Table 1. While the nature of the framework allows virtually all material properties to be determined from the literature, either via experiments or *ab-initio* simulations, we provide, where necessary, additional remarks on the selection of specific parameters employed in our simulations.

Table 1. Material properties for modeling the electro-chemo-mechanical behavior of the Si anode – LPSC electrolyte system pulled from literature[7,11–18] and this work.

	Parameter	Value	Source
**Chemical**	D_0	$1.47 \cdot 10^{-12}$ m²/s	[11, 12]
	$\Omega c_{R,max}$	2.625	[13]
	$c_{R,max}$	$0.295 \cdot 10^6$ mol/m³	[14]
	$[a_2 - a_7]/F$	[0.8735,0.7185, −4.504,6.876 − 4.6272,1,1744] V	[15]
**Elastic**	E_{a-Si}	80 GPa	[16]
	ν_{a-Si}	0.22	[16]
	E_{Li}	4.91 GPa	[16]
	ν_{Li}	0.36	[16]
	E_{LPSC}	22 GPa	[17]
	ν_{LPSC}	0.37	[17]
**Plastic**	$\dot{\varepsilon}_0$	$2.3 \cdot 10^{-3} s^{-1}$	[18]
	n	2.94	[18]
	Y_0	1.6 GPa	[7]
	Y_{sat}	0.4 GPa	[7]
	$\bar{c}_*$	0.04	[7]
**Fracture**	ζ	1.0 MPa · s	This work

	$\psi^{*}\ell$	7.2 – 10 $\mathrm{J/m^2}$	[19]
	ℓ	2.0 μm	Regularization Parameter

We vary the elastic properties of the Si-anode with extent of lithiation, making use of the standard rule of mixtures relation,

$$E = aE_{Li} + (1-a)E_{Si} \tag{20}$$

$$\nu = a\nu_{Li} + (1-a)\nu_{Si} \tag{21}$$

with,

$$a = \frac{x_{max}\cdot\bar{c}}{1 + x_{max}\cdot\bar{c}} \tag{22}$$

Here, $\{E_{Li},\, \nu_{Li}\}$ represent the elastic properties of pure lithium, while $\{E_{Si},\, \nu_{Si}\}$ the elastic properties of pure silicon. Additionally, a denotes the atomic fraction of lithium atoms, while x_{max} is the maximum stochiometric amount of lithium in the Li_xSi compound. We assume that silicon may be fully lithiated to the compound $Li_{15}Si_4$, so that $x_{max} = 3.75$. The elastic moduli $\{E, \nu\}$ are then converted to $\{G, K\}$ moduli for use in (11) and (12), making use of the standard relations $G = E/\big(2(1+\nu)\big)$ and $K = E/\big(3(1-2\nu)\big)$.

As detailed in Eq. 14, ζ introduces a viscous regularization parameter to our model, serving to impart stability to our numerical solution scheme. We note here that choice of a sufficiently small ζ is done so that it has a negligible effect on the simulation results across the various cases considered in this work, while imparting stability to our solver. The selected value of the viscous regularization parameter for the model at hand is also consistent with similar works in the literature.[8]

Additionally, consistent with the discussion of Eq. 14, ψ^* introduces a dissipated energy per unit volume during the fracture process, while ℓ accounts for the damage gradient effects in our formulation. As discussed by Narayan and Anand[19], the gradient damage formulation will be mesh-independent, provided the characteristic element size (h_e) is taken to be sufficiently small compared to the length scale parameter (ℓ). Typically, an element size $h_e \leq 0.2 \cdot \ell$ is considered sufficient. Additionally, the product $\psi^*\ell$ can be physically related to the macroscopic critical energy release rate of the material of choice $(\mathcal{G}_c)$ through,[8,19]

$$\mathcal{G}_c \approx \psi^*\ell \tag{23}$$

Provided the length scale parameter (ℓ) is chosen in a suitable physically realistic range, ℓ acts then as an adjustable regularization parameter in our gradient damage theory. With ℓ fixed, the value of ψ^* can be subsequently derived such that $\mathcal{G}_c \approx \psi^*\ell$ holds. We prescribe a length scale (ℓ) of 2 μm for our model at hand, based on which a characteristic element size (h_e) of 400 nm is prescribed for our meshing needs. This choice of element size accordingly satisfies the criterion $h_e \leq 0.2 \cdot \ell$ for mesh independence.

As a final remark regarding our theoretical formulation, we note that the coupling of species diffusion to mechanics in Eq. 19 is omitted in our simulations for computational amenability. That is, we revisit Eq. 19 to reflect $\mu = \mu_0 + R\vartheta \ln\left(\gamma \frac{\bar{c}}{1-\bar{c}}\right)$. In Sect. IV below, we elaborate further on this choice and show through simulation that it has a negligible effect on the results.

III. **Finite Element Modeling**

The theoretical framework discussed in Sections I and II above is implemented in the finite-element package Abaqus / Standard through the development of a custom finite element (UEL),

which allows us to solve the governing equations associated with the three degrees of freedom: 1) displacement, 2) chemical potential, and 3) damage.

The finite element analysis simulations were performed on a portion of the silicon/LPSC half-cell under plane-strain conditions. The simulation domain is shown schematically in Fig. S15a; note that we do not show the full height of the LPSC. The width (distance AD) of the simulation domain is 150 µm, the initial height of the silicon (h_0) (distance AB) is 10 µm (unless otherwise noted), and the initial height of the LPSC substrate (distance BC) is 300 µm. Nodes along the left edge (edge AC) and the right edge (edge DF) are prescribed zero horizontal displacement. Consistent with experiments, a 10 MPa stack pressure is applied on the top edge of the silicon film (edge AD). The interface between the silicon and LPSC electrolyte (edge BE) is perfectly bonded. We employ 375 elements across the width (AD), 25 elements across the height of the silicon (AB), and 20 elements across the height of the LPSC (BC). All elements are quadrilateral, fully integrated, plane-strain elements.

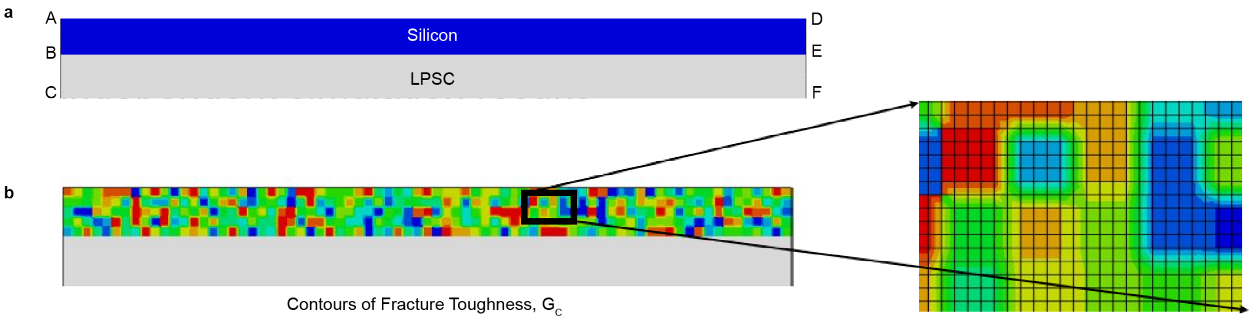

Figure S15. (a) FEA simulation domain. (b) FEA simulation domain showing contours of initial fracture toughness distribution.

A constant flux (current), determined by a desired C-rate, is prescribed on the silicon/LPSC interface (edge BE). The applied flux is related to C-rate through the simple relation,

$$j_{applied} = -\left(V/A_{surf}\right) \cdot c_{max} \cdot (c_{rate}/3600) \tag{24}$$

where V is the original volume of the silicon electrode, A_{surf} is the original area over which the flux is applied, and c_{max} is the maximum molar concentration of lithium in the silicon-lithium alloy as listed in Table 1.

During the lithiation half-cycle, flux is linearly ramped from an initial value of zero to a final value ($j_{applied}$) at $t_{ramp\ lithiation}$, at which point it is held constant for a time ($t_{lithiation}$). Subsequently, during the delithiation half-cycle, flux is linearly reversed over a span ($t_{ramp\ delithiation}$) and then held constant until the end of the simulation (t_{end}).

We account for inherent microstructural heterogeneities (i.e. defects, voids etc.) in the silicon film prior to electrochemical cycling through a spatially varying uniform distribution of fracture toughness (G_c) across the anode (see Fig. S15b). As shown on the inset of Fig S15b, we chose a spatial defect size of 2 μm by 2 μm and randomly assigned a fracture toughness from the distribution. The distribution is centered around a mean $\overline{G_c} = 8.6\, J/m$ and uniformly varied over a range $\pm$ 20%.

IV. Diffusion stress-coupling modification

As detailed in Section 1 above, the chemical potential for diffusion if Li in the solid is traditionally coupled to stress through Eq. (19), via,

$$\mu = \mu_0 + R\vartheta \ln\left(\gamma \frac{\bar{c}}{1-\bar{c}}\right) - g(\mathrm{d})\frac{1}{3}\mathrm{tr}(\mathbf{M}^e) \tag{25}$$

Numerically, the coupling between the chemical potential (μ) and damage (d) adds significant numerical difficulties, and reduces convergence, as both of these are solution variables in the finite element method. As such we chose to remove the stress-coupling and implement a chemical potential of the form,

$$\mu = \mu_0 + R\vartheta \ln\left(\gamma \frac{\bar{c}}{1-\bar{c}}\right) \tag{26}$$

Figure S16 shows contours of damage (top row), concentration (middle row), and the horizontal stress component (bottom row) for two simple simulations in which we do not have heterogeneity in the underlying fracture toughness. As such, a single dominant crack is formed during delithiation of the silicon. Importantly, Fig. S16a (left column) shows a stress-coupled simulation with chemical potential of the form Eq. 25 above, while Fig. S16b (right column) shows a stress-uncoupled simulation with chemical potential of the form Eq. 26 above. We can observe that the simulations are nearly identical, predicting virtually the same damage, concentration, and stress fields. This illustrates that removal of the coupling between chemical potential and stress has negligible effect in the simulations considered in this manuscript. Thus, the chemical potential and stress were uncoupled for the simulations to improve computational time.

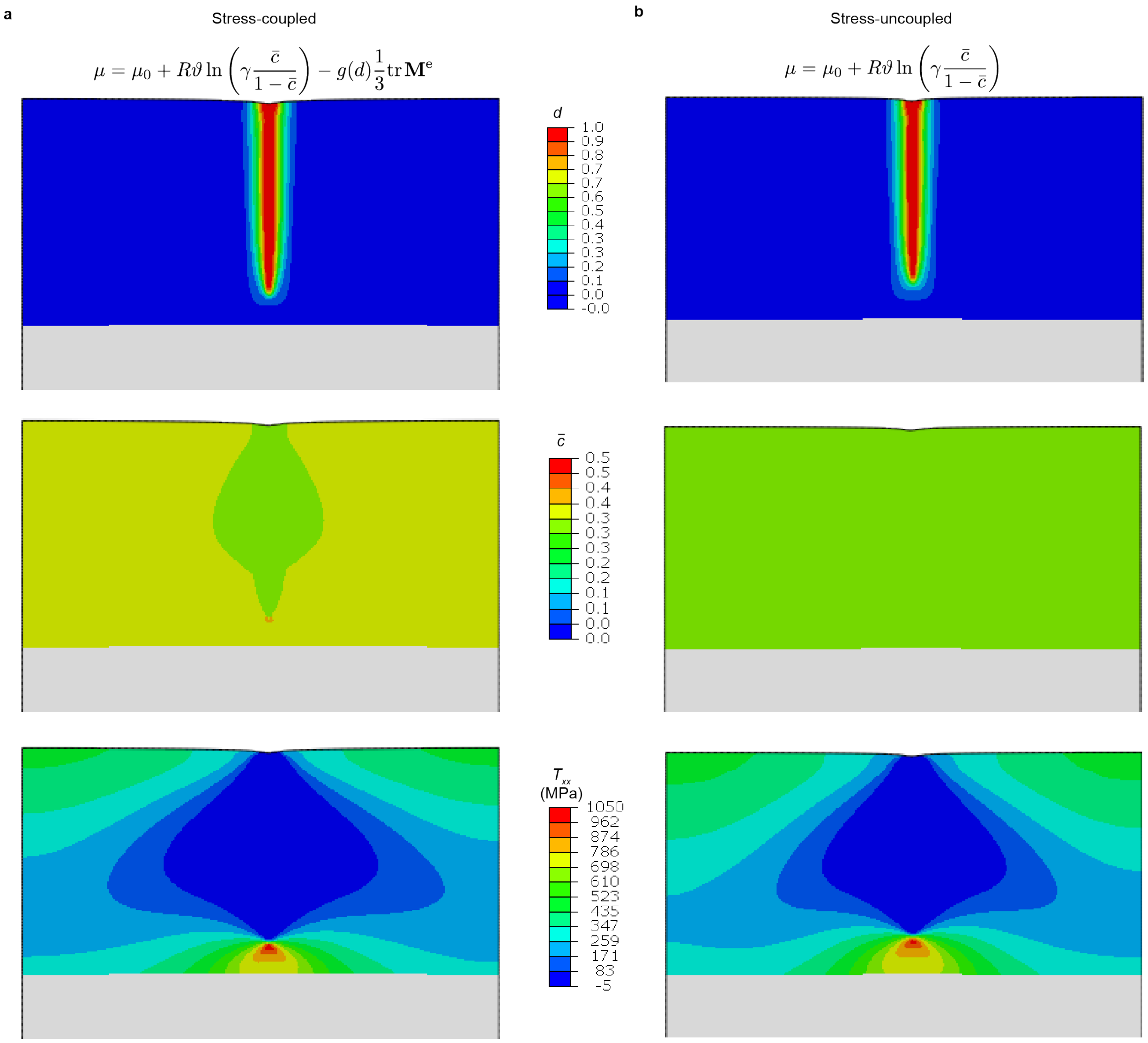

Figure S16. Contours of damage, concentration, and horizontal stress during delithiation of a homogeneous (no imperfections) silicon electrode. (a) Employs a stress-coupled chemical potential while (b) employs a stress-uncoupled chemical potential.

Section S5 – Flat Interface Simulations

As described in the main text of this manuscript, we first consider the evolution of damage in a silicon/LPSC system containing a flat, uniform interface. Figure S17 shows the spatial distribution of fracture toughness across the simulated silicon films (left), and the corresponding

simulated crack patterns (right). Further, in Fig. S17 we show three simulations having varied initial toughness distribution with initial silicon thickness (h_0) of 10 µm and three simulations with initial film thickness of 6 µm. Average crack spacing in a given simulation is computed by measuring the approximate space between cracks internal to the simulation domain as shown on the right column of Fig. S17. We can again observe that geometry, such as film thickness, plays an important role in determining average crack spacing, while the initial distribution of defects does not.

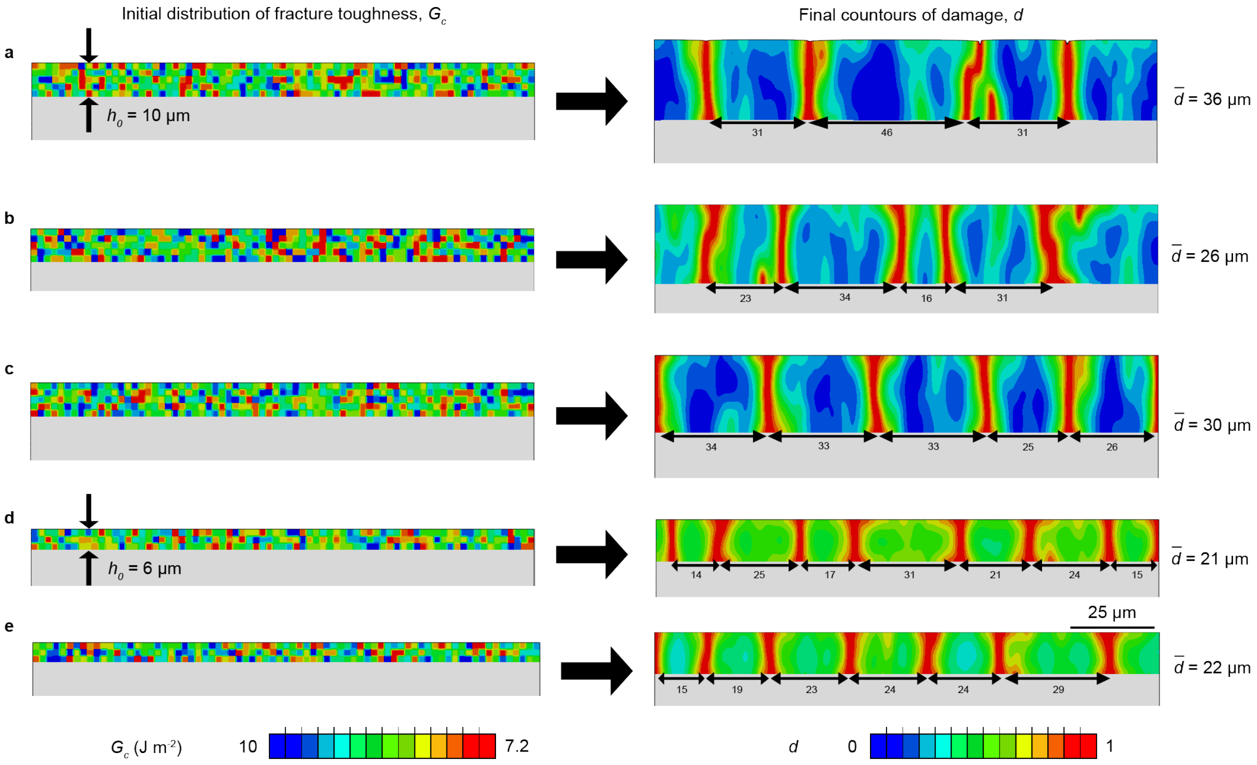

Figure S17. Initial distribution in fracture toughness (left) and resulting final crack pattern (right). The top three rows are simulations with an initial thickness (h_0) of 10 µm, while the bottom two rows have an initial thickness of 6 µm. Arrows between cracks on the right-hand side were used to compute the average crack spacing in a given simulation ($\bar{d}$).

For completeness, Fig. S18 shows contours of (a) concentration, (b) horizontal stress, and (c) damage during delithiation of a flat interface silicon / LPSC simulation domain corresponding to Fig. 5a in the main portion of this manuscript.

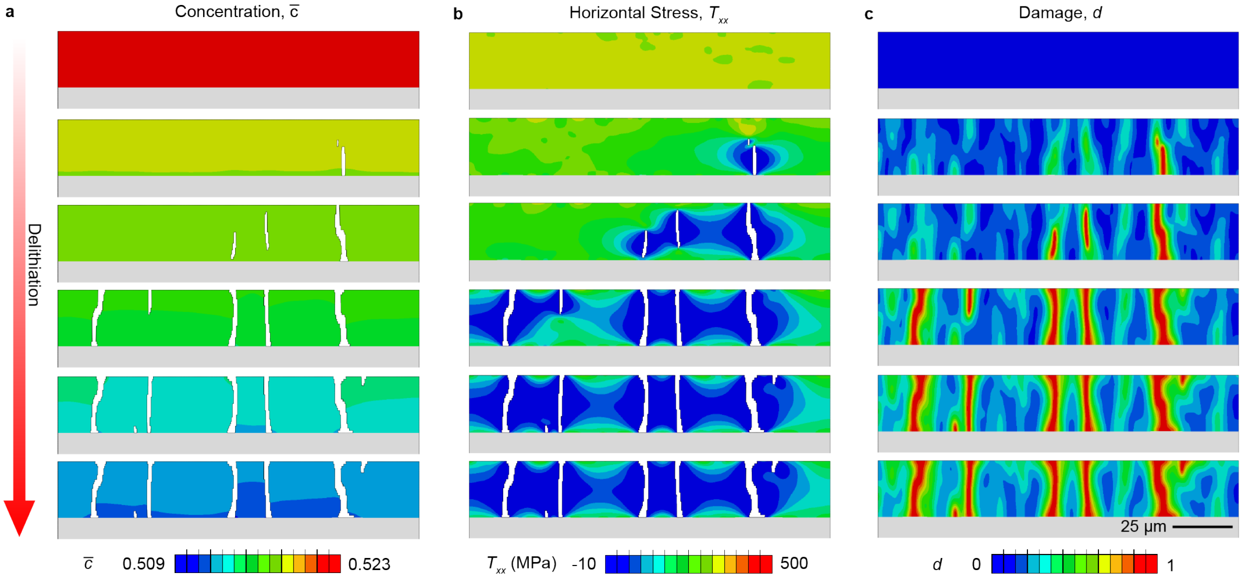

Figure S18. Contours of (a) concentration, (b) horizontal stress, and (c) damage corresponding to Fig. 6 in the main manuscript. Elements with $d > 0.95$ are hidden in the contours of concentration and horizontal stress to illustrate crack formation and propagation.

Section S6 – Rough interface simulations

Now we consider a silicon/LPSC system in which the interface is subjected to a surface imperfection. Figure 5b in the main manuscript shows the resulting formation of both vertical cracks and horizontal delamination cracks in the model system which grow underneath the imperfection. Importantly, delamination (i.e. horizontal cracks) can occur during delithiation either underneath the imperfection (as shown in Fig. 3b) or away from the imperfection. This phenomenon is governed by the stress state in the material after lithiation. If we consider a film which is stress-free in the pristine state, then during lithiation we will accumulate significant compressive stresses after lithiation. This is illustrated in Fig. S19, where (a) shows contours of vertical stress at the start of delithiation. We can observe here large compressive stresses

underneath the imperfection arise due to the lithiation induced volume increase in this confined configuration. Some of these stresses are released (relaxed) due to plastic flow, however significant compressive stresses remain. During delithiation of such a system, vertical tensile stresses concentrate away from the imperfection (Fig. S19b) which result in delamination occurring away from the imperfection (Fig. S19c).

In contrast, we consider a simulation which is stress-free in the lithiated state, as shown in the right column of Fig. S19. Here (d) shows the contours of vertical stress at the start of delithiation, which as expected are zero given that this is now the stress-free configuration. At the start of delithiation (Fig. S19e) we now observe the formation of tensile stresses underneath the imperfection, leading to the concentration of delamination underneath the imperfection (Fig. S19f). The numerical results in the right column of Fig. S19 qualitatively match the experimental observations shown in Figure 3b of the main manuscript. This suggests that in experiments there is significant stress relaxation during the first lithiation half-cycle.

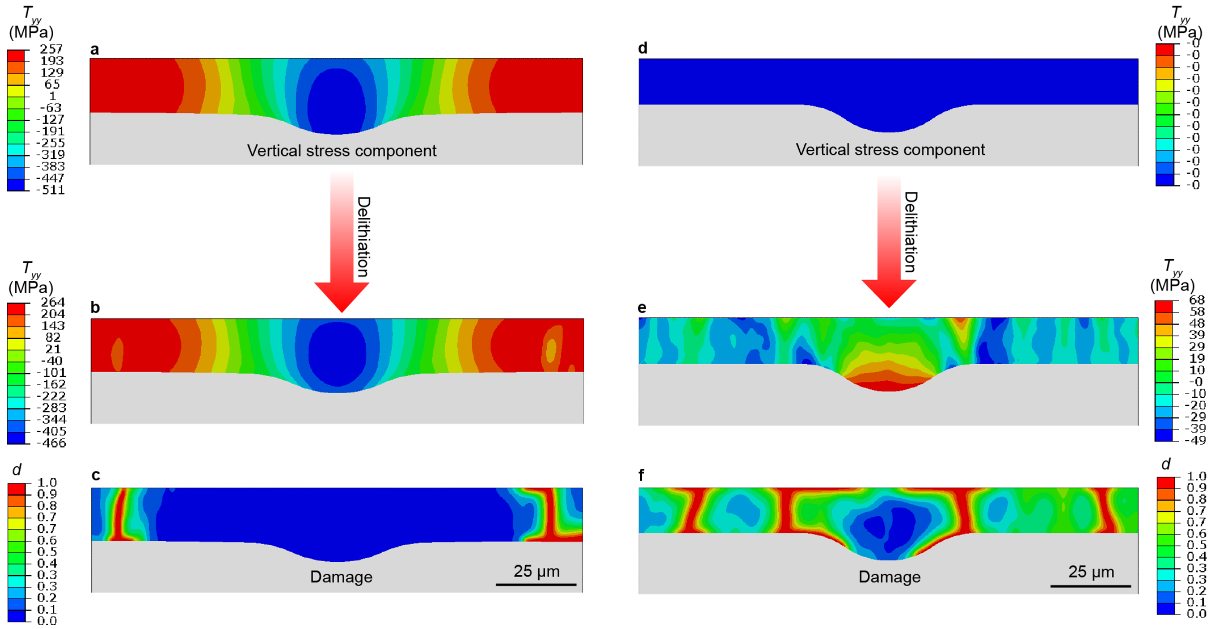

Figure S19. Comparison of simulations with different initial stress-free configurations. The left column (a-c) is stress-free in the pristine configuration (i.e., compressive stress exists in the protrusion after lithiation), and the right column (d-f) is stress-free in the lithiated configuration. (a) and (d) show contours of vertical stress at the start of delithiation (lithiated configuration), while (b) and (e) show contours early during delithiation. (c) and (f) show contours of damage at the end of the simulation.

Finally, we note that the simulations presented in Fig. S19 are run with a smaller total volumetric expansion. Specifically, we use half the partial molar volume listed in Table 1 above. The smaller partial molar volume significantly improves numerical convergence while maintaining the qualitative nature of the results observed.

Section S7 – Sensitivity Analysis

We consider here the sensitivity of the simulations to a few critical material parameters. First, in Fig. S20 we show simulations with varying lithium diffusivity. The left column shows contours of concentration and damage for the baseline value of $D_0 = 1.47 \cdot 10^{-12}\ m^2/s$. The right column shows two simulations with the diffusivity decreased by one and two orders of

magnitude. As expected, we can observe in the contours of concentration that larger gradients in concentration arise as we decrease the diffusivity. The overall nature of the fracture pattern, however, remains consistent, and we observe no meaningful change in the pattern. It is expected that at much lower diffusivities large concentration gradients will develop which will in turn affect the stress state in the material and potentially the fracture pattern.

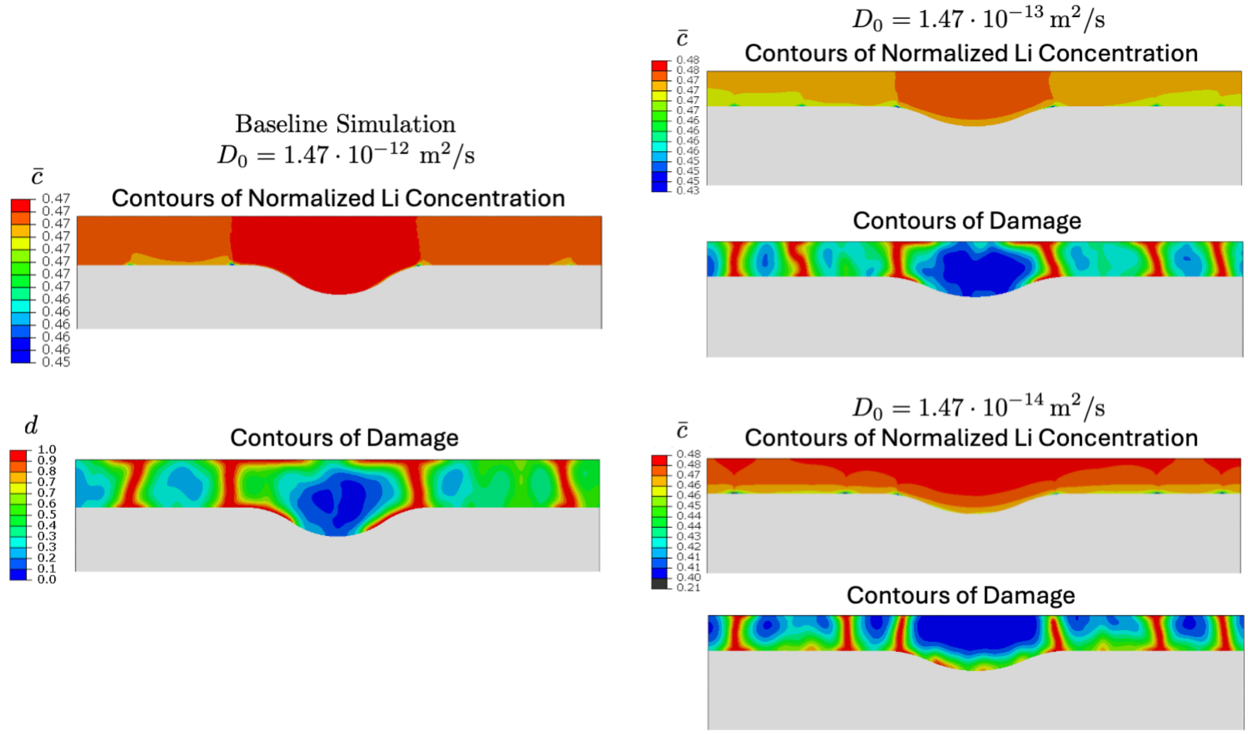

Figure S20. Simulations with decreasing diffusivity coefficient.

Next, we consider the sensitivity to plasticity. In Fig. S21 we compare the baseline simulation having a yield strength at saturation of $Y_{sat} = 0.4\ GPa$ to a new simulation having yield strength of $Y_{sat} = 0.2\ GPa$, with all other simulation parameters remaining the same. As shown in Fig. S21, the reduction in yield strength does not affect the fracture pattern or average crack spacing. It does, however, affect when fracture initiates. As we lower the yield strength, we allow for

more of the elastic stresses generated during lithiation/delithiation to relax. As such, fracture occurs later during the delithiation process when significant delithiation induced stress has been generated to lead to fracture. This is shown in Fig. S21, where the simulation with lower yield strength experiences full fracture at a later stage when the film has undergone significant delithiation and is thus thinner.

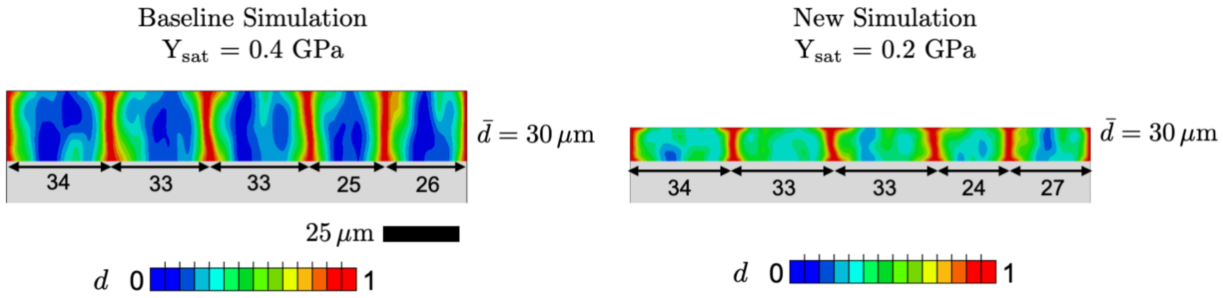

Figure S21. Simulations with decreased plastic yield strength.

Finally, we consider the sensitivity of our simulations to the mean fracture toughness. As shown in Fig. S22, we performed two additional simulations with increased average fracture toughness while preserving the same distribution (left column in Fig. S22). Fig S22a shows the baseline simulation with an average toughness of $G_c = 8.6\, J/m^2$; this is increased to $G_c = 12.6\, J/m^2$ in Fig. S22b and $G_c = 16.6\, J/m^2$ in Fig S22c.

As shown in Fig. S22, as we increase the average fracture toughness, we do not see a significant change in the fracture pattern and average crack spacing. Comparing Figs. S22a and S22c, we can observe a delay in the formation of cracks marked by the thinning of the film during

delithiation. This is expected due to the increased toughness requiring more build up of elastic stresses to generate fracture.

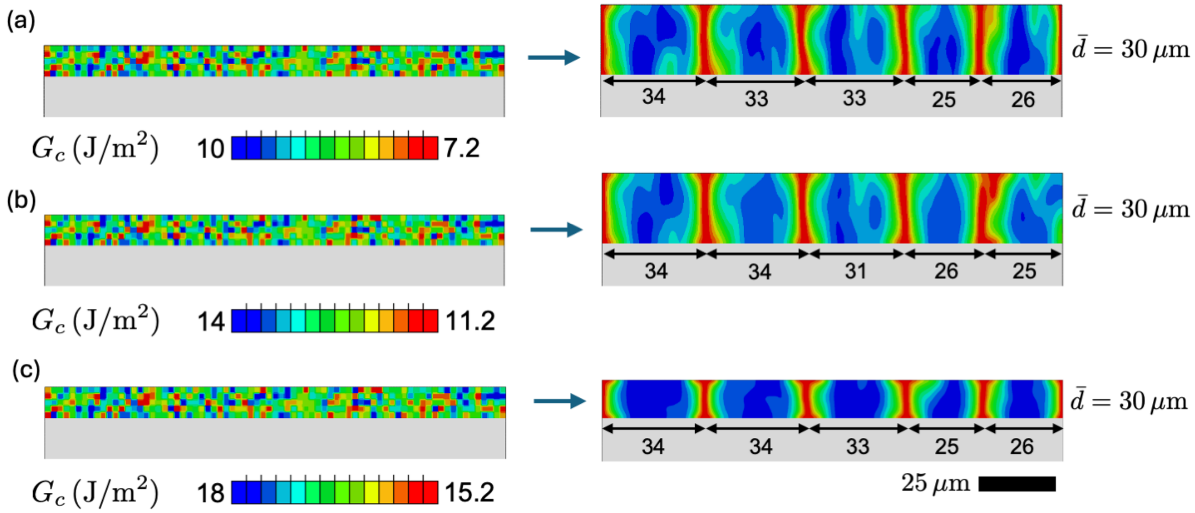

Figure S22. Simulations with increased average fracture toughness.

SUPPLEMENTARY VIDEO CAPTIONS

Video S1. Top-down XCT video of an image sliced of the silicon electrode in the *operando* XCT experiment during delithiation (1-4 s) and relithiation (5-7 s). During delithiation, the formation of the "mud pattern" channel crack network is observed, while during relithiation the crack network partially disappears.

Video S2. Top-down views of the segmented crack network in the silicon electrode in the same orientation as Video S1 during delithiation (1-4 s) and relithiation (5-7 s). Because other voxels are removed, crack voxels throughout the entire silicon electrode volume are visible. During delithiation, the formation of the same "mud pattern" channel crack network in Video S1 is observed with the addition of large interfacial cracks spanning beneath entire silicon domains, separating them from the LPSC SSE. During relithiation, both channel cracks and interfacial cracks partially disappear while some new interfacial cracks appear beneath previously connected silicon domains.

Video S3. Time-series of cross-sectional XCT image slices shown in Fig. 3a showing the delithiation (1-4 s) and relithiation (5-7 s) of a region of the silicon electrode with relatively uniform thickness. Vertical channel cracks form during delithiation and then partially close during relithiation.

Video S4. Time-series of cross-sectional XCT image slices in Fig. 3b showing the delithiation (1-4 s) and relithiation (5-7 s) of a region of the silicon electrode with non-uniform thickness that

exhibits delithiation-driven interfacial fracture. Vertical channel cracks and a large crack at the Li_xSi/SSE interface form during delithiation and then mostly close during relithiation

Video S5. Time-series of cross-sectional XCT image slices in Fig. 3c showing the delithiation (1-4 s) and relithiation (5-7 s) of a region of the silicon electrode with non-uniform thickness that exhibits relithiation-driven interfacial fracture. Vertical channel cracks and a small crack at the Li_xSi/SSE interface form during delithiation. During relithiation, the vertical channel cracks mostly close while the small interfacial crack grows wider as the relatively large silicon domain is completely separated from the LPSC SSE.

Video S6. Time-series of phase-field modeling simulations in Fig. 5 showing the concentration (top row), stress (middle row), and damage (bottom row) evolution of the flat Li_xSi/SSE interface in Fig. 5a (left) and the rough Li_xSi/SSE interface in Fig. 5b (right) during delithiation of a Li_xSi electrode. Interfacial damage is predicted to occur underneath the rough interface due to greater stress at the rough interface as compared to the flat interface.

SUPPLEMENTARY REFERENCES

(1) Han, S. Y.; Lee, C.; Lewis, J. A.; Yeh, D.; Liu, Y.; Lee, H. W.; McDowell, M. T. Stress Evolution during Cycling of Alloy-Anode Solid-State Batteries. *Joule* **2021**, *5* (9), 2450–2465. https://doi.org/10.1016/j.joule.2021.07.002.

(2) Lewis, J. A.; Cortes, F. J. Q.; Liu, Y.; Miers, J. C.; Verma, A.; Vishnugopi, B. S.; Tippens, J.; Prakash, D.; Marchese, T. S.; Han, S. Y.; Lee, C.; Shetty, P. P.; Lee, H. W.; Shevchenko, P.; De Carlo, F.; Saldana, C.; Mukherjee, P. P.; McDowell, M. T. Linking Void and Interphase Evolution to Electrochemistry in Solid-State Batteries Using Operando X-Ray Tomography. *Nat. Mater.* **2021**, *20* (4), 503–510. https://doi.org/10.1038/s41563-020-00903-2.

(3) Lewis, J. A.; Sandoval, S. E.; Liu, Y.; Nelson, D. L.; Yoon, S. G.; Wang, R.; Zhao, Y.; Tian, M.; Shevchenko, P.; Martínez-Pañeda, E.; McDowell, M. T. Accelerated Short Circuiting in Anode-Free Solid-State Batteries Driven by Local Lithium Depletion. *Adv. Energy Mater.* **2023**, *13* (12), 2204186. https://doi.org/10.1002/aenm.202204186.

(4) Gürsoy, D.; De Carlo, F.; Xiao, X.; Jacobsen, C. TomoPy: A Framework for the Analysis of Synchrotron Tomographic Data. *J. Synchrotron Radiat.* **2014**, *21* (5), 1188–1193. https://doi.org/10.1107/S1600577514013939.
(5) Obrovac, M. N.; Christensen, L. Structural Changes in Silicon Anodes during Lithium Insertion/Extraction. *Electrochem. Solid-State Lett.* **2004**, *7* (5), A93–A96. https://doi.org/10.1149/1.1652421.
(6) Obrovac, M. N.; Christensen, L.; Le, D. B.; Dahn, J. R. Alloy Design for Lithium-Ion Battery Anodes. *J. Electrochem. Soc.* **2007**, *154* (9), A849–A855. https://doi.org/10.1149/1.2752985.
(7) Di Leo, C. V.; Rejovitzky, E.; Anand, L. Diffusion–Deformation Theory for Amorphous Silicon Anodes: The Role of Plastic Deformation on Electrochemical Performance. *Int. J. Solids Struct.* **2015**, *67–68*, 283–296. https://doi.org/10.1016/j.ijsolstr.2015.04.028.
(8) Anand, L.; Mao, Y.; Talamini, B. On Modeling Fracture of Ferritic Steels Due to Hydrogen Embrittlement. *J. Mech. Phys. Solids* **2019**, *122*, 280–314. https://doi.org/10.1016/j.jmps.2018.09.012.
(9) Miehe, C.; Hofacker, M.; Welschinger, F. A Phase Field Model for Rate-Independent Crack Propagation: Robust Algorithmic Implementation Based on Operator Splits. *Comput. Methods Appl. Mech. Eng.* **2010**, *199* (45), 2765–2778. https://doi.org/10.1016/j.cma.2010.04.011.
(10) Bistri, D.; Di Leo, C. V. A Continuum Electro-Chemo-Mechanical Gradient Theory Coupled with Damage: Application to Li-Metal Filament Growth in All-Solid-State Batteries. *J. Mech. Phys. Solids* **2023**, *174*, 105252. https://doi.org/10.1016/j.jmps.2023.105252.
(11) Chen, L. B.; Xie, J. Y.; Yu, H. C.; Wang, T. H. An Amorphous Si Thin Film Anode with High Capacity and Long Cycling Life for Lithium Ion Batteries. *J. Appl. Electrochem.* **2009**, *39* (8), 1157–1162. https://doi.org/10.1007/s10800-008-9774-1.
(12) Obrovac, M. N.; Krause, L. J. Reversible Cycling of Crystalline Silicon Powder. *J. Electrochem. Soc.* **2006**, *154* (2), A103–A108. https://doi.org/10.1149/1.2402112.
(13) Mohr, P. J.; Taylor, B. N. CODATA Recommended Values of the Fundamental Physical Constants: 1998. *Rev. Mod. Phys.* **2000**, *72* (2), 351–495. https://doi.org/10.1103/RevModPhys.72.351.
(14) Bucci, G.; Nadimpalli, S. P. V.; Sethuraman, V. A.; Bower, A. F.; Guduru, P. R. Measurement and Modeling of the Mechanical and Electrochemical Response of Amorphous Si Thin Film Electrodes during Cyclic Lithiation. *J. Mech. Phys. Solids* **2014**, *62*, 276–294. https://doi.org/10.1016/j.jmps.2013.10.005.
(15) Sethuraman, V. A.; Chon, M. J.; Shimshak, M.; Van Winkle, N.; Guduru, P. R. In Situ Measurement of Biaxial Modulus of Si Anode for Li-Ion Batteries. *Electrochem. Commun.* **2010**, *12* (11), 1614–1617. https://doi.org/10.1016/j.elecom.2010.09.008.
(16) Deng, Z.; Wang, Z.; Chu, I.-H.; Luo, J.; Ong, S. P. Elastic Properties of Alkali Superionic Conductor Electrolytes from First Principles Calculations. *J. Electrochem. Soc.* **2015**, *163* (2), A67–A74. https://doi.org/10.1149/2.0061602jes.
(17) Pharr, M.; Suo, Z.; Vlassak, J. J. Variation of Stress with Charging Rate Due to Strain-Rate Sensitivity of Silicon Electrodes of Li-Ion Batteries. *J. Power Sources* **2014**, *270*, 569–575. https://doi.org/10.1016/j.jpowsour.2014.07.153.

(18) Pharr, M.; Suo, Z.; Vlassak, J. J. Measurements of the Fracture Energy of Lithiated Silicon Electrodes of Li-Ion Batteries. *Nano Lett.* **2013**, *13* (11), 5570–5577. https://doi.org/10.1021/nl403197m.
(19) Narayan, S.; Anand, L. A Gradient-Damage Theory for Fracture of Quasi-Brittle Materials. *J. Mech. Phys. Solids* **2019**, *129*, 119–146. https://doi.org/10.1016/j.jmps.2019.05.001.
